# Early pulmonary response is critical for extra-pulmonary carbon nanoparticle mediated effects: comparison of inhalation versus intra-arterial infusion exposures in mice

**DOI:** 10.1186/s12989-017-0200-x

**Published:** 2017-06-20

**Authors:** Koustav Ganguly, Dariusch Ettehadieh, Swapna Upadhyay, Shinji Takenaka, Thure Adler, Erwin Karg, Fritz Krombach, Wolfgang G. Kreyling, Holger Schulz, Otmar Schmid, Tobias Stoeger

**Affiliations:** 1 Unit of Lung and Airway Research, Institute of Environmental Medicine (IMM), Karolinska Institutet, SE-171 77 Stockholm, Sweden; 2 Unit of Work Environment Toxicology, Institute of Environmental Medicine (IMM), Karolinska Institutet, SE-171 77 Stockholm, Sweden; 3 Institute of Lung Biology and Disease, Comprehensive Pneumology Center, Helmholtz Zentrum München, German Research Center for Environmental Health, D85764 Neuherberg, Germany; 4 German Mouse Clinic, Institute of Experimental Genetics, Helmholtz Zentrum München, German Research Center for Environmental Health, D85764 Neuherberg, Germany; 5 Cooperationgroup Comprehensive Molecular Analytics (CMA), Joint Mass Spectrometry Centre (JMSC), Helmholtz Zentrum München, German Research Center for Environmental Health, D85764 Neuherberg, Germany; 6 Walter Brendel Centre of Experimental Medicine, Ludwig-Maximilians-Universität, D81377 Munich, Germany; 7 Institute of Epidemiology I, Helmholtz Zentrum München, German Research Center for Environmental Health, D85764 Neuherberg, Germany; 8 Comprehensive Pneumology Center Munich (CPC-M), Member of the German Center for Lung Research, D85764 Munich, Germany

**Keywords:** Inhaled soot, Ultrafine particulate matter, Inflammation, Lung, Heart, Aorta, Cardiovascular, Particle translocation

## Abstract

**Background:**

The death toll associated with inhaled ambient particulate matter (PM) is attributed mainly to cardio-vascular rather than pulmonary effects. However, it is unclear whether the key event for cardiovascular impairment is particle translocation from lung to circulation (direct effect) or indirect effects due to pulmonary particle-cell interactions. In this work, we addressed this issue by exposing healthy mice via inhalation and intra-arterial infusion (IAI) to carbon nanoparticles (CNP) as surrogate for soot, a major constituent of (ultrafine) urban PM.

**Methods:**

Equivalent surface area CNP doses in the blood (30mm^2^ per animal) were applied by IAI or inhalation (lung-deposited dose 10,000mm^2^; accounting for 0.3% of lung-to-blood CNP translocation). Mice were analyzed for changes in hematology and molecular markers of endothelial/epithelial dysfunction, pro-inflammatory reactions, oxidative stress, and coagulation in lungs and extra-pulmonary organs after CNP inhalation (4 h and 24 h) and CNP infusion (4 h). For methodological reasons, we used two different CNP types (spark-discharge and Printex90), with very similar physicochemical properties [≥98 and ≥95% elemental carbon; 10 and 14 nm primary particle diameter; and 800 and 300 m^2^/g specific surface area] for inhalation and IAI respectively.

**Results:**

Mild pulmonary inflammatory responses and significant systemic effects were observed following 4 h and 24 h CNP inhalation. Increased retention of activated leukocytes, secondary thrombocytosis, and pro-inflammatory responses in secondary organs were detected following 4 h and 24 h of CNP inhalation only. Interestingly, among the investigated extra-pulmonary tissues (i.e. aorta, heart, and liver); aorta revealed as the most susceptible extra-pulmonary target following inhalation exposure. Bypassing the lungs by IAI however did not induce any extra-pulmonary effects at 4 h as compared to inhalation.

**Conclusions:**

Our findings indicate that extra-pulmonary effects due to CNP inhalation are dominated by indirect effects (particle-cell interactions in the lung) rather than direct effects (translocated CNPs) within the first hours after exposure. Hence, CNP translocation may not be the key event inducing early cardiovascular impairment following air pollution episodes. The considerable response detected in the aorta after CNP inhalation warrants more emphasis on this tissue in future studies.

**Electronic supplementary material:**

The online version of this article (doi:10.1186/s12989-017-0200-x) contains supplementary material, which is available to authorized users.

## Background

Epidemiological studies have linked both short-term and long-term particulate matter (PM) exposures to increased morbidity and mortality [1–4]. The World Health Organization (WHO) estimated over 800,000 premature deaths worldwide per year to be attributed to PM air pollution [5]. Cardiovascular impairments have been identified by several epidemiological studies as a major PM associated health risk accounting for more fatalities than pulmonary effects [6–10]. Ultrafine or nano-sized carbon particles (UfCP; diameter less than 100 nm) originating from incomplete combustion processes (soot) have been hypothesized as the major PM constituent contributing of cardiovascular effects [11] (mainly because of their: 1) greater pulmonary deposition and retention efficiency in the lung periphery compared to larger sized particles [12, 13]; 2) enhanced oxidant capacity [14, 15]; 3) higher pulmonary pro-inflammatory potential [16, 17]; and 4) higher efficiency to penetrate the epithelium to reach interstitial sites or even translocate to the systemic circulation and secondary organs (such as brain, heart, aorta, liver etc.) [18–24].

Exposure to urban UfCPs has increased dramatically over the last decades primarily due to the technological revolution resulting in increased emissions of combustion-derived ultrafine particles (soot) from traffic, industry, and heating activities [17]. Consequently, the major constituent of urban ultrafine particles is soot, i.e. carbonaceous material consisting of an elemental carbon core surrounded by various types of organic carbon and small fractions of metal, sulfate, and nitrate compounds [25, 26]. Elemental carbon nanoparticles (CNPs; primary particle diameter ~ 25 nm) constitute the core of combustion derived particles [27] and represent a relevant toxicological surrogate for exhaust particles from diesel engines, if particle surface area is used as dose metric [28, 29]. Motor-vehicle emissions consist of a complex mixture of particulate, chemical and gaseous pollutants such as fine particulate matter (PM2.5; diameter < 2.5 μm), ultrafine particles (UFPs; diameter < 0.1 μm), metals, volatile organic material, black carbon, ozone etc. [30]. A major component of urban PM air pollution is CNPs. They constitute the core of combustion derived particles [27] and represents relevant surrogates for exhaust particles from modern diesel engines [28, 29]. Increased use of engineered nanoparticles has made CNP as an evolving source of human exposure [17]. However, it is to be considered that engineered CNPs and ambient UfCPs in spite of their similar sizes (<100 nm) are likely to have different modes of action, but may also share certain common modes of action, particularly those driven by the elemental carbon core.

Plausible mechanisms of UfCP-mediated cardiovascular effects have been addressed in many studies. We have previously shown that exposing young and healthy mice to high number concentrations of CNPs induces local acute inflammatory effects in the lung [31]. Furthermore, we could reproduce the epidemiological findings of PM-related cardiovascular risks in an experimental setting by exposing spontaneously hypertensive rats (SHRs) with ambient dust and CNPs [30, 32, 33]. We detected not only blood pressure changes and heart rate variability, but our findings also indicated promotion of endothelial dysfunction and thrombogenesis as the plausible causes for cardiovascular impairments triggered by pulmonary CNP deposition [34, 35]. Others have shown that CNP inhalation causes acute vascular effects in healthy and asthmatic subjects, indicated by changes in peripheral blood leukocyte distribution and adhesion molecule expression [36, 37].

It is generally assumed that PM exposure elicits low-grade systemic inflammation and subsequent changes in vascular function, such as increased blood leukocyte and platelet counts, and fibrinogen levels which in turn can result in cardiovascular impairments [38]. Therefore, endothelial dysfunction, inflammation, pro-inflammatory cytokine imbalance, oxidative stress, and dysregulated coagulation pathways may be considered as the triggering events of cardiovascular impairments due to particulate air pollution. Several hypotheses have been put forward to explain the mechanisms through which inhaled particles can interact with the cardiovascular system [8, 39]. The classical concept is that inhaled particles locally evoke an inflammatory response in the lung with subsequent releases of pro-inflammatory and/or pro-thrombotic mediators into the circulation [11]. In addition to this indirect effect on circulation and extra-pulmonary organs, more recent studies suggest that particularly inhaled insoluble nanoparticles (but not micron-sized particles) are capable of rapid translocation into the circulation [21, 40] thereby affecting hemostasis and cardiovascular integrity by direct CNP interaction with circulation and extra-pulmonary organs [41, 42]. In this direct mode of action, the lungs act as an efficient port of entry of the primary toxin (CNPs in this case), while in the indirect mode of action the lungs act as mediator or even amplifier of a local (pulmonary) inflammatory response yielding systemic effects. The direct mode of action is supported by the findings of Nurkiewicz and colleagues [43] who reported systemic microvascular responses in rats following pulmonary exposure to residual oil fly ash and TiO_2_ independent of the degree of pulmonary inflammation. In another more direct approach, we have shown that a moderate dose of 10^7^ intra-arterially infused CNPs induced platelet accumulation in the hepatic microvasculature of mice [34]. This was associated with pro-thrombotic changes on the endothelial surface of hepatic microvessels. Similarly, inhalation of CNPs evoked platelet adhesion and fibrinogen deposition in mice at corresponding doses [35]. However, the key question that still remains to be answered; i.e. whether the extra-pulmonary effects following inhalation of insoluble particles result from molecular events initiated in the lungs (indirect mode) or translocation of particles beyond the lungs (direct mode) drives the extra-pulmonary effects, or both.

The overarching aim was to investigate, if extra-pulmonary effects of CNP inhalation can be explained by direct interactions of lung-blood translocated particles on extra-pulmonary tissue (i.e. heart, liver, aorta) or if the local particle-lung cell interaction in lung is the key event. To pursue this, we have compared pulmonary and systemic effects for two routes of CNP application at equivalent dose, namely CNP application directly into the blood stream (intra-arterial infusion/IAI) and pulmonary application (inhalation) with likely subsequent translocation of a small CNP fraction into the blood stream. BALB/cJ mice were assessed after 4 h and 24 h inhalation exposure, or 4 h after IAI. Time points were selected based on our previous studies showing that in lungs, CNPs induced a two-phase inflammatory response after 4 or 24 h inhalation [31], whereas the extra-pulmonary pro-coagulatory impact in the hepatic microvasculature was detected as early as 2 h after CNP IAI application [34, 35]. Therefore, we sought to systematically and independently investigate the two potential modes of action by applying (i) similar CNPs, as used in several of our previous studies [44–48] and (ii) equivalent doses of CNP in mice via inhalation or intra-arterial infusion (IAI) and subsequent comparison of the effects in the lung, blood, and secondary target organs such as heart, aorta, and liver.

## Results

Our analysis strategy involved different body compartments namely, bronchoalveolar space, lung tissue, blood (cells and plasma), heart, liver and aorta thereby providing a comprehensive assessment of each compartment in terms of endothelial/epithelial activation, inflammation, and oxidative stress, on the protein and gene expression level. According to the current understanding and as previously outlined by Kermanizadeh et al. [49], oxidative stress, inflammation, cytotoxicity and dysfunction of cellular/physiological processes present the likely causal mechanism of toxicological effects in secondary organs following inhalation and eventually translocation of nanoparticles. All data discussed here are compared to the corresponding time-matched control and are statistically significant (*p* < 0.05). In case of transcript or protein expression data, which was generated from pooled tissue samples, we have only discussed molecules with ≥2 fold increased or decreased expression levels [Additional file 1: Table S1].

To sum up the events matching to experimental design timings: The pro-inflammatory response was detectable at the protein level already after the first 4 h of CNP inhalation exposure. It is plausible that the pro-inflammatory mediators are released from, i) intracellular stores (vesicles) [50]; or ii) extracellular stores (matrix immobilized) [51]. However neutrophil recruitment to the lungs and development of a pro-inflammatory gene expression signature might take longer time. Thus, a moderate but significant pro-inflammatory gene signature is detected after a 24 h CNP inhalation. Whereas in case of IAI, pro-inflammatory protein and gene signature is completely absent at 4 h even if the applied IAI dose is equivalent to the calculated translocated amount within 24 h of inhalation. Therefore, we believe, by avoiding the 24 h IAI time point we implemented the reduction of animal use principle of the “3R–tenet” without significantly compromising scientific quality. Our experimental strategy was also based on our previous experience where we observed that in case of IAI the time point of 2 h was sufficient to develop platelet – endothelial interactions [34]. Hence, we strategized to investigate at 4 h after IAI of CNP allowing an additional 2 h for further downstream effects. The 4 h time point investigated in this study is also based on the fact that platelet – endothelial interactions have also been detected already 2 h after CNP inhalation and effects did not increase 8 h thereafter [34].

### Pulmonary response

**Fig. 1:**
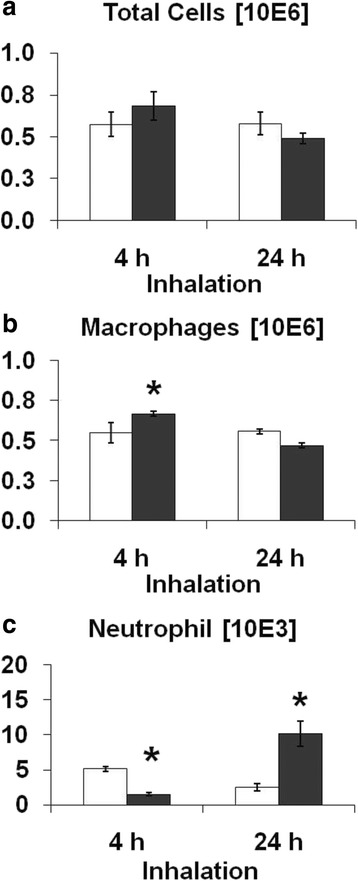
Analysis of bronchoalveolar lavage (BAL) cell differentials following carbon nanoparticle (CNP) inhalation exposure in BALB/cJ mice compared to control. **a**. Total BAL cell counts do not exhibit any significant changes. BAL cell analysis following CNP inhalation exposed mice revealed increased macrophages (**b**) [Control: 5.5 ± 0.8 × 10^5^ versus 6.7 ± 0.8 × 10^5^] at 4 h post exposure followed by a strong granulocyte influx (**c**) [Control: 2.6 ± 0.8 × 10^3^ versus 10.0 ± 2.8 × 10^3^] at 24 h post exposure. Data are shown as Mean ± SEM; *n* = 8 and asterisks (*) denote *p* < 0.05. *White bars*: Clean air exposed; *Black bars*: CNP exposed

In line with previous results [30], CNP inhalation caused a moderate, acute inflammatory response in the lungs. Accordingly, BAL fluid analysis revealed a mild but significant neutrophilic granulocyte influx resulting in 10.2 ± 2.5 × 10^3^ neutrophils (versus 2.5 ± 0.8 × 10^3^ in controls) in the CNP exposed group after 24 h inhalation [Fig. 1; Additional file 1: Table S2]. BAL protein concentration was increased after 4 h CNP inhalation and returned to basal levels after 24 h inhalation [Additional file 1: Table S3]. The analysis of BAL cytokine levels revealed an initially decreased concentration of CXCL1 (2.6 fold) after 4 h inhalation, while CXCL1 was increased 4.5 fold over control levels after 24 h CNP inhalation, which is in agreement with elevated BAL neutrophil numbers. Other more monocyte/macrophage, but not directly to granulocyte recruitment related cytokines such as CCL2, IL1a and IL12p40 showed non-elevated levels in the CNP exposed animals [Additional file 1: Table S3] thereby supporting the mild nature of the inflammation.

**Fig. 2:**
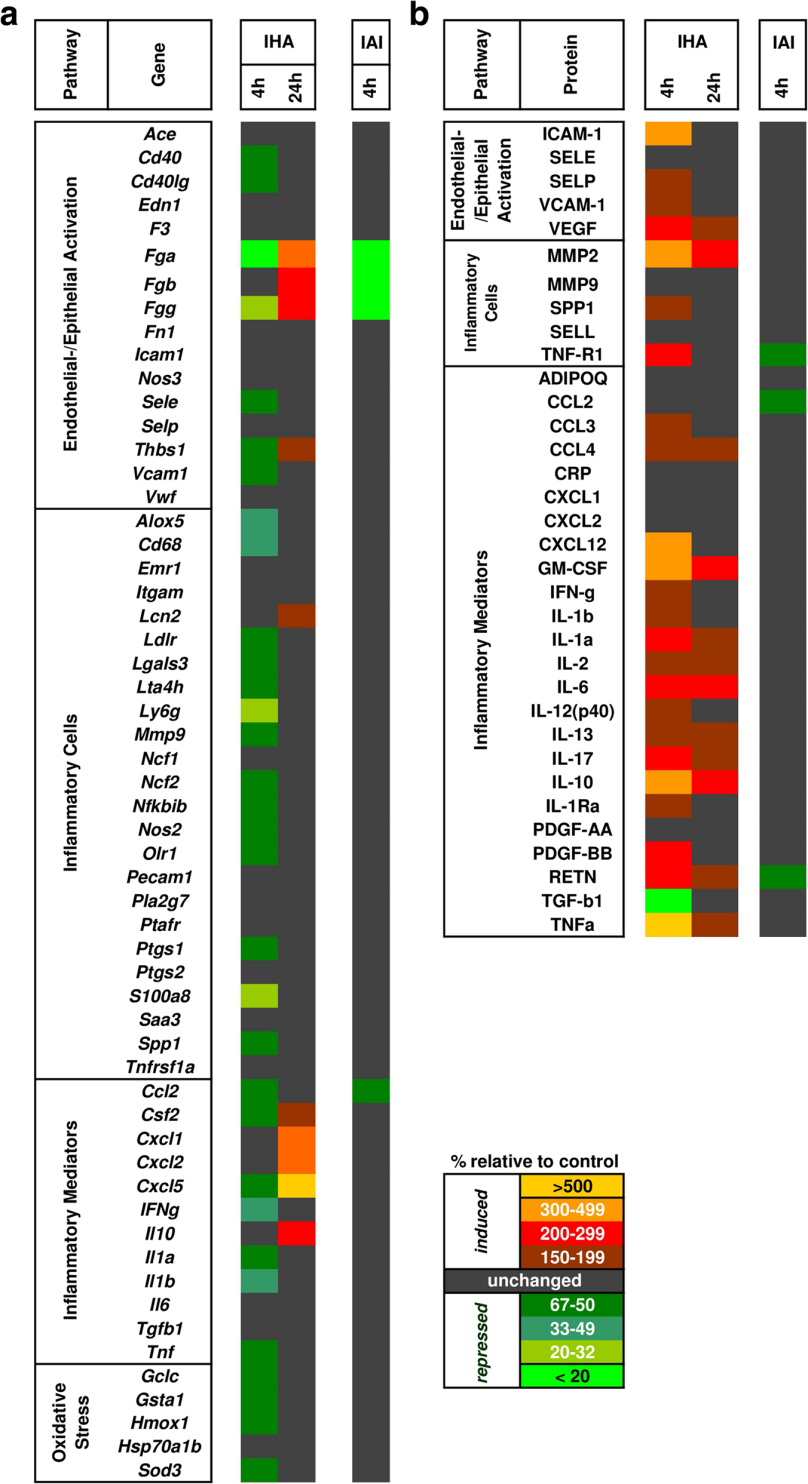
Heatmap representation of lung transcript and protein panel assays following inhalation and intra-arterial infusion exposure of carbon nanoparticles (CNP). **a.** Transcript expression levels of 59 genes representing epithelial/endothelial activation, inflammatory cell markers, inflammation mediators and oxidative stress are shown. **b.** Protein expression levels of 34 markers representing epithelial/endothelial activation, inflammatory cell markers, inflammation mediators are shown. Samples were pooled from 4 animals/experimental group for transcript and protein analysis. Expression values are provided as percentage relative to time matched control. Fold changes below 1.5 were considered insignificant and are indicated in *black color*. IHA: inhalation; IAI: intra-arterial infusion

Transcript expression analysis of 59 genes representative of endothelial/epithelial dysfunction, pro-inflammatory reactions, oxidative stress, and coagulation in the lung tissue was performed to characterize the response pattern after inhalation or IAI of CNPs [Fig. 2a, Additional file 1: Table S4]. As shown by the BAL data, CNP inhalation caused a pro-inflammatory signature in lung tissue extracts with elevated levels of fibrinogen (*Fga*, −b, −g) and cytokine (*Il10, Cxcl1, −2, −5*) transcript expression after 24 h CNP inhalation as well as high abundance of proteins associated with inflammation and endothelial/epithelial activation after 4 h and 24 h CNP inhalation (Fig. 2a, b). In contrast to inhalation, 4 h after CNP IAI no pro-inflammatory changes could be detected in the lungs, indicating that this route of exposure effectively bypassed an inflammatory stimulation of the lung tissue within the 4 h time window of investigation. Hence, we conclude that IAI application of CNPs resulted in direct extra-pulmonary effects only, while inhalation of CNPs may have resulted in both direct pulmonary and indirect extra-pulmonary effects.

### Blood response

**Fig. 3:**
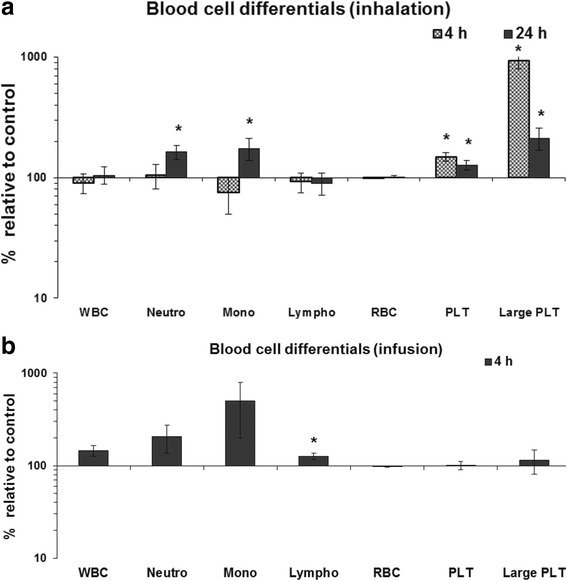
Hematological analysis of blood samples following inhalation and intra-arterial infusion of carbon nanoparticle (CNP) in mice compared to control. CNP exposure related changes are shown as percentage relative to control. **a.** Granulocyte and monocyte numbers were increased in the blood following 24 h CNP inhalation exposure. [Granulocytes: 0.46 ± 0.09 versus 0.75 ± 0.05 × 10E3; Monocytes: 0.04 ± 0.01 versus 0.07 ± 0.01 × 10E3 cells/ml blood]. Platelet counts, particularly that of large platelets, were significantly increased both after 4 h and 24 h post exposure periods. [Platelet (4 h post exposure): 810 ± 87 versus 1203 ± 55; Platelet (24 h post exposure): 869 ± 97 versus 1104 ± 18 and Large platelet (4 h post exposure): 1.9 ± 0.4 versus 17.6 ± 2.7; Large platelet (24 h post exposure): 4.3 ± 1.3 versus 9.0 ± 1.3 x10E3 cells/ml blood]. **b.** Intra-arterial infusion of CNP resulted in increased lymphocyte counts after 4 h [Lymphocyte: 1.52 ± 0.07 versus 1.92 ± 0.13 x10E3 cells/ml blood] but the platelet count remained unaltered. Data are shown as Mean ± SEM; inhalation: *n* = 8, infusion: *n* = 6 and asterisks (*) denote *p* < 0.05. WBC: White blood cell; Neutro: neutrophilic granulocytes; Mono: Monocytes; Lympho: Lymphocytes; RBC: Red blood cells; PLT: Platelets; Large PLT: Large platelets

Hematological analysis revealed increased granulocyte (1.6-fold) and monocyte (1.8-fold) counts after 24 h CNP inhalation exposure indicating a mild inflammatory, systemic reaction (Fig. 3a, b; Table 1; Additional file 1: Table S5). Following IAI of CNP, only a mild increase in lymphocyte counts (1.3-fold) was detected. Total platelet count was slightly increased by 1.5-fold after 4 h and 1.3-fold after 24 h of CNP inhalation (Fig. 3a, Table 1, Additional file 1: Table S5). Interestingly, only post CNP inhalation the quantity of large platelets was strongly affected showing a > 9-fold (at 4 h) and >2-fold (at 24 h) increase. This suggests that 4 h pulmonary exposure to CNP initiated a reactive thrombocytosis which persisted through 24 h of CNP inhalation. Thrombocytosis is considered as a predisposing event to atherosclerotic plaque formation. Recently, Tabor et al. [52] reported accelerated arterial thrombus formation in rat models following diesel exhaust particle exposure due to increased platelet activation independent of pulmonary and systemic inflammation or impaired fibrinolytic function. Increase of large platelets indicates the formation of new platelets thereby increasing the total platelet count. Large platelets are also associated with platelet activation during hemostasis. In accordance, also other platelet indices characterizing platelet volume and granularity (e.g. MPV, PTC, MPC, PCDW and MPM) were altered both after 4 h and 24 h CNP inhalation (Table 1). These findings all together support the notion of platelet activation after CNP inhalation. In contrast, platelet count, platelet shape, and platelet activity parameters remained unchanged following IAI (Fig. 3b, Table 1, Additional file 1: Table S5). The recent pioneering work from Lefrançais et al. [53] demonstrated lung as an important site of local platelet biogenesis and a potent reservoir for haematopoietic progenitors. In light of their findings [53], it is plausible that local, pulmonary stress-response to particle inhalation may eventually via local reactive oxygen species (ROS) formation [54] directly lead to rapid increases of blood platelet numbers and thereby increase the risk for cardiac infarction without involving further systemic cytokine elevations and bone marrow activation.

**Table 1:** Hematological analysis of blood samples for platelet parameters following inhalation (4 h and 24 h) and intra-arterial infusion (4 h) of carbon nanoparticle (CNP) in mice compared to control

Platelet Parameters	Inhalation						Intra-arterial Infusion		
	4 h			24 h			4 h		
	Control	CNP	p	Control	CNP	p	Control	CNP	p
PLT (×10E3 CELLS/μL)	809.50 ± 86.95	1203.13 ± 54.84	*↑	868.50 ± 96.65	1103.57 ± 17.77	*↑	902.50 ± 62.56	909.67 ± 69.40	NS
Large PLT (×10E3 CELLS/μL)	1.88 ± 0.40	17.63 ± 2.71	*↑	4.25 ± 1.32	9.00 ± 1.34	*↑	22.33 ± 4,72	25.50 ± 5.60	NS
MPV (fl)	5.45 ± 0.15	7.66 ± 0.16	*↑	5.99 ± 0.29	7.04 ± 0.10	*↑	7.97 ± 0.30	8.28 ± 0.58	NS
PCT (%)	0.45 ± 0.06	0.92 ± 0.04	*↑	0.53 ± 0.08	0.78 ± 0.02	*↑	0.73 ± 0.07	0.74 ± 0.06	NS
PDW (%)	51.65 ± 1.15	58.70 ± 0.79	*↑	53.63 ± 2.37	56.99 ± 1.02	NS	68.83 ± 2.52	65.25 ± 1.70	NS
MPC (g/dl)	24.93 ± 0.42	19.86 ± 0.32	*↓	23.53 ± 0.72	21.29 ± 0.21	*↓	19.90 ± 0.58	19.58 ± 1.12	NS
PCDW (g/dl)	7.41 ± 0.17	8.10 ± 0.08	*↑	7.61 ± 0.24	8.21 ± 0.06	*↑	7.95 ± 0.10	7.93 ± 0.11	NS
MPM (pg)	1.23 ± 0.01	1.26 ± 0.01	*↑	1.24 ± 0.01	1.27 ± 0.01	*↑	1.31 ± 0.01	1.31 ± 0.01	NS
PMDW (pg)	0.43 ± 0.01	0.42 ± 0.01	NS	0.43 ± 0.01	0.43 ± 0.01	NS	0.49 ± 0.01	0.48 ± 0.01	NS

*PLT* platelet, *Large PLT* large platelet count, *MPV* mean platelet volume, *PCT* plateletcrit, *PDW* platelet distribution width, *MPC* mean platelet component, *PCDW* Platelet component distribution width, *MPM* mean platelet mass, *PMDW* platelet mass distribution width. Data is shown as Mean ± SEM; inhalation: *n* = 8; infusion: *n* = 6; * *p* < 0.05; ↑: significantly increased; ↓: significantly decreased; *NS* not significant

**Fig. 4:**
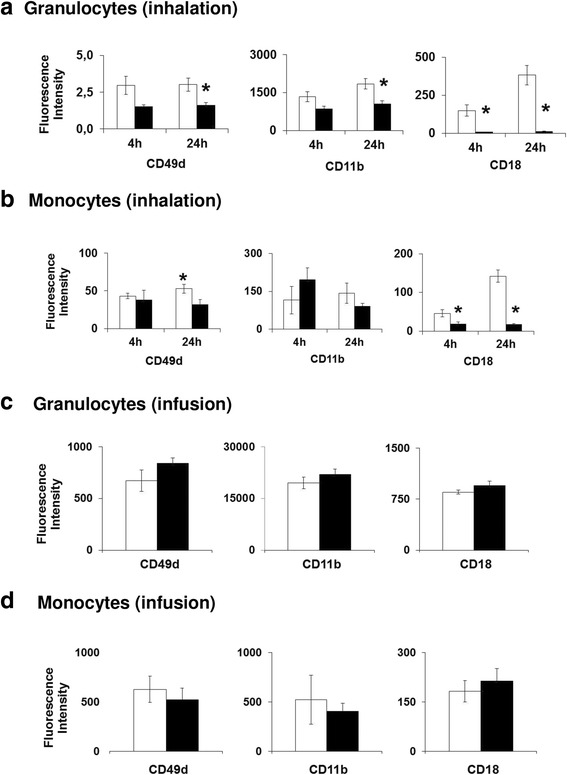
Characterization of peripheral blood monocytes and leucocytes for surface expression of adhesion molecules following inhalation and intra-arterial infusion of carbon nanoparticle (CNP) in mice compared to control using fluorescent automated cell sorting (FACS). **a.** Reduced expression of CD49d and CD11b was noted in granulocytes after 24 h of CNP inhalation exposure whereas CD18 expression was reduced in granulocytes at both 4 h and 24 h post inhalation exposure. **b.** Reduced expression of CD49d in monocytes was observed after 24 h of CNP inhalation exposure, while reduced CD18 expression in monocytes was noted following both 4 h and 24 h post CNP inhalation exposure. **c.** No altered expression of CD49d, CD11b and CD18 was noted in granulocytes following 4 h of intra-arterial infusion of CNP. **d.** No altered expression of CD49d, CD11b and CD18 was noted in monocytes following 4 h of intra-arterial infusion of CNP. Data are shown as Mean ± SEM; inhalation: *n* = 8, infusion: *n* = 6; and asterisks (*) denote *p* < 0.05. *White bars*: Clean air exposed; *Gray bars*: CNP exposed

In view of the observed changes in blood neutrophil and monocyte numbers and following the human exposure study from Frampton and colleagues [36], we additionally investigated the surface expression of the adhesion molecules CD11b, CD18 and CD49d to assess the activation status of these circulating leukocytes. Similar to the findings of Framton et al. [36] in human, reduced surface expression of CD49d, CD11b (24 h) and CD18 (4 h) was noted on granulocytes after CNP inhalation (*p* < 0.05; *n* = 8). Also for monocytes a reduced CD18 expression was observed after both time points (Fig. 4 a, b). No effect, however, was observed following IAI (Fig. 4 c, d). Reduced surface expression of β1 and β2 integrin leukocyte adhesion molecules CD11b, CD18 and CD49d in peripheral blood monocytes and granulocytes particularly 24 h post inhalation may indicate an impeding effect on peripheral blood leukocyte distribution, arguing for an increased retention of activated cells in the pulmonary vascular bed due to CNP inhalation. In this context, an inflammatory activation of monocytes and neutrophils in the vascular bed of CNP exposed lungs, with increased expression of CD11b and CD18 integrins, is likely to decrease cell deformability via actin polymerization, again contributing to leukocyte retention and reduced capillary flow [55]. As mentioned before, very similar responses have previously been described in CNP-exposed healthy and asthmatic subjects [36]. In this study also a reduction in pulmonary diffusing capacity for carbon monoxide in CNP exposed individuals was observed, additionally supporting the hypothesis of Frampton and colleagues, of a mismatch in ventilation and pulmonary vascular perfusion and pulmonary capillary flow caused by CNP inhalation.

**Fig. 5:**
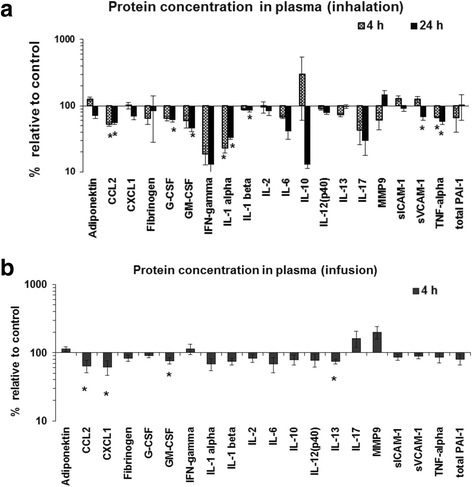
Expression profiling of acute phase reactants and systemic inflammation markers in the plasma of carbon nanoparticle (CNP) exposed mice compared to control following inhalation and intra-arterial infusion. **a.** CNP exposure related changes of protein concentrations are shown as percentage relative to control following 4 h and 24 h post inhalation is shown. **b.** CNP exposure related changes of protein concentrations are shown as percentage relative to control following 4 h post intra-arterial infusion. Data are presented as Mean ± SEM; inhalation: *n* = 8, infusion: *n* = 6; and asterisks (*) denote *p* < 0.05

A panel of 25 protein analytes known to be associated with cardiovascular diseases consisting of acute phase reactants, soluble inflammatory adhesion molecules, inflammation related proteinases, and cytokines were screened in the plasma (Additional file 1: Table S6). Only the plasma levels of the cytokines GM-CSF (2.3 fold) and IL1α (4.6 fold) were more than 2-fold reduced following CNP inhalation after 4 and 24 h CNP exposure (*p* < 0.05; *n* = 8; Fig. 5a). No difference equaling or exceeding a 2-fold change could be detected following IAI of CNPs (Fig. 5b). Noteworthy, the concentrations of some cytokines and acute phase reactants, i.e. fibrinogen, PAI-1, CXCL1, G-CSF, and IL-6, but also CCL2 and CCL5 were markedly higher in controls of the IAI groups compared to the inhalation scenario, which may indicate an acute phase response caused by the surgical procedure rather than CNP exposure and, thus, may limit the interpretation of these specific endpoints. Yet other cytokines in particular IL-1a and - b, known to show up early during an inflammatory response, remain unchanged between inhalation and IAI controls.

### Transcript and protein analysis in extra-pulmonary organs

**Fig. 6:**
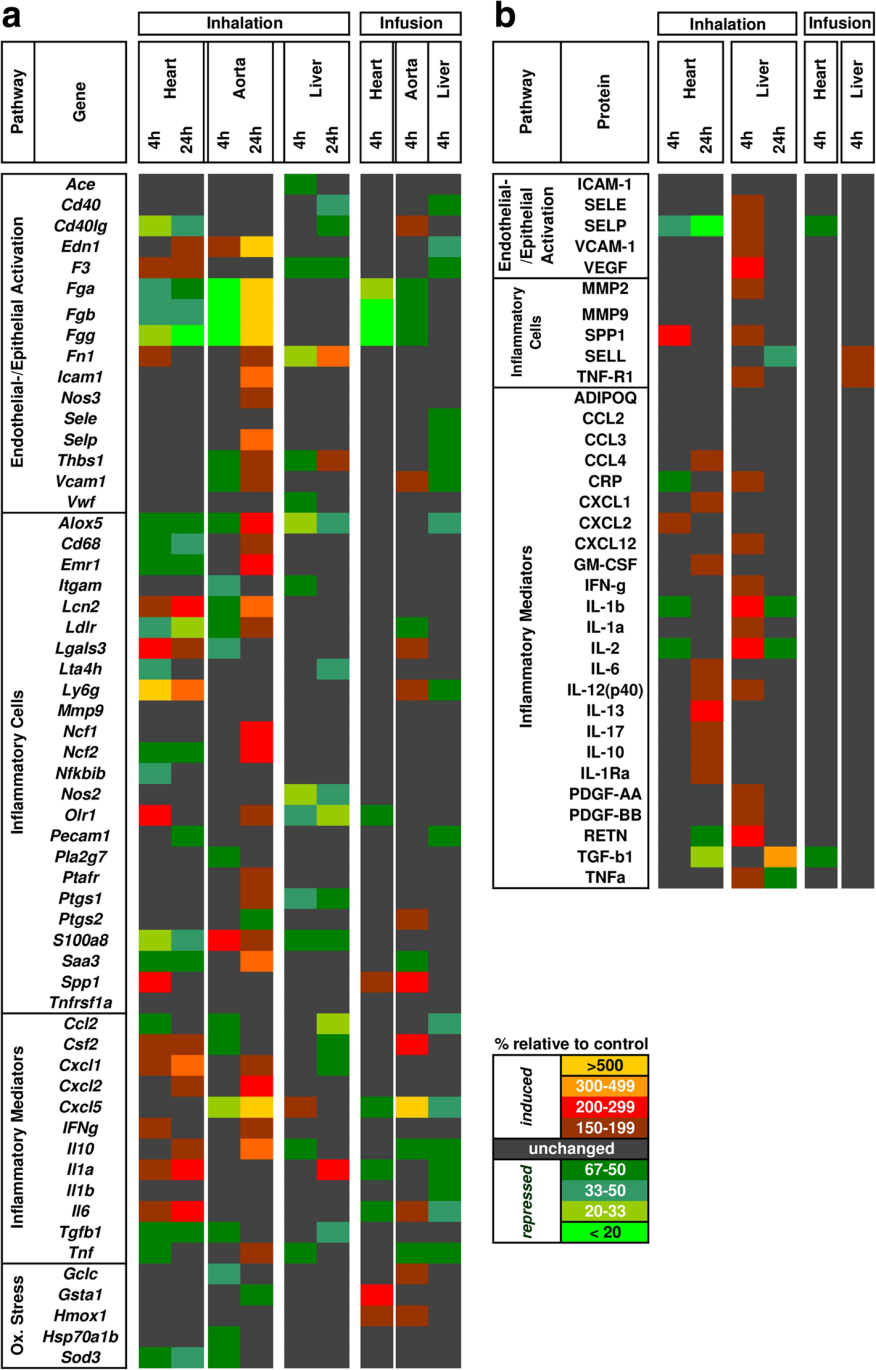
Heatmap representation of the transcript (59 genes) and protein (34 analytes) panel profiling from heart, aorta and liver following carbon nanoparticle (CNP) inhalation (4 h and 24 h) and intra-arterial infusion (4 h) comparing exposed versus control mice. **a.** Transcript expression levels of 59 genes representing epithelial/endothelial activation, inflammatory cell markers, inflammation mediators and oxidative stress of heart, aorta and liver are shown. **b.** Protein expression levels of 34 markers representing epithelial/endothelial activation, inflammatory cell markers, inflammation mediators of heart and liver are shown. Samples were pooled from 4 animals/experimental group for transcript and protein analysis. Expression values are provided as percentage relative to time matched control. Fold changes below 1.5 were considered insignificant and are indicated in black color. Ox. stress: Oxidative Stress

Transcript and protein panel assays were carried out in heart, aorta, and liver tissue (Fig. 6; Additional file 1: Table S7) to assess and compare the effect of CNP exposure via inhalation and IAI routes. After CNP inhalation transcript analysis of the heart detected several inflammatory cell markers, such as *Lcn2, Lgals3, Ly6g, Olr1* and *Spp1* as well as the cytokines *Cxcl1, Il1a* and *Il6* to be elevated (Fig. 6). In contrast, only the antioxidant response gene *Gsta1* was more than 2-fold induced by IAI. Similarly, liver extracts showed increased levels for inflammation-related transcripts *Fn1* and *Il1a* after CNP inhalation exposure only. Aorta samples, in fact, revealed the most striking pro-inflammatory RNA signature with 15 markers upregulated after 24 h of CNP inhalation suggesting endo/epithelial activation and inflammatory leucocyte accumulation. Again, only 3 genes were upregulated after IAI exposure. A similar pattern was observed for the protein profile, with several pro-inflammatory proteins induced after CNP inhalation in heart (24 h) and liver (4 h) samples (SPP1, IL-1b, IL-2, IL-13, RETN and TGF-b1), but none after infusion (Fig. 6). Due to limitations in sample weights, aortas were only assessed for mRNA but not protein.

## Discussion

Realizing the broad spectrum of the experiments performed in this work and the corresponding data generated following analysis of pulmonary and extra-pulmonary body compartments, we have explained several aspects of the findings along with the results. Summarizing the events, our findings indicate that equivalent doses of translocated CNP following inhalation - when intra-arterially infused - do not cause appreciable extra-pulmonary effects. Correspondingly, CNP inhalation induces a very mild inflammatory response in lung tissue with profound systemic effects. Among the studied target organs, the aorta appears to exhibit a high susceptibility to CNP. Increased transcript levels of neutrophil-related chemoattractants (*Cxcl2, −5; Lcn2*) and macrophage markers (*Ncf1, −2; Saa3, Emr1*) indicate plausible accumulation of leukocytes in the aorta. Further, elevated levels of fibrinogens (*Fga, −b, −g*) suggests triggered homeostatic response and blood leukocyte adhesion. Increased transcript levels of markers associated with atherosclerosis or cardiovascular impairments (*Edn1, Selp, Alox5,* and *Icam1*) further indicate plausible detrimental effects of CNP exposure on aortic tissue at 24 h post inhalation. Increased levels of *Alox5, Emr1, Cxcl2, Saa3, Ncf1* and *Ncf2* may also suggest inflammatory phagocyte accumulation in the aorta for 24 h particle inhalation. These findings are supported by the acute protein response in the liver, after 4 h inhalation and in hematologic parameters, particularly platelet numbers as well as a decline in circulating activated leucocytes. In spite of the fact that the observed pattern in this study matches well with the previously described platelet accumulation and fibrinogen deposition in the extra-pulmonary microvasculature after CNP inhalation [35], these findings are at this point only indicative of atherosclerotic susceptibility and warrant detailed mechanistic investigations to substantiate a link to disease onset or progression. Recently, Miller and colleagues [56] demonstrated the accumulation of inhaled gold nanoparticles (<10 nm) at the sites of vascular inflammation following translocation in a size dependent manner. This finding provides new evidence of a plausible contribution of translocated nanoparticles in the pathogenesis of atherosclerosis and cardiovascular diseases. In our IAI study however, where an inflammatory response in the lungs was bypassed and the retention of activated blood leukocytes was absent, much less thrombocytosis was observed, and the extra pulmonary organs of investigation failed to show a pro-inflammatory signature. To match that to the accumulation data from Miller and colleagues [56], it might be considered that we used young and healthy mice, with now vascular lesions, and that particles, smaller than 10 nm may have more profound effects on extra-pulmonary organs. Nevertheless our study indicates the aortic tissue to be a susceptible organ for extra-pulmonary particle effects.

Taken together, our data shows that CNP inhalation induced a very mild inflammatory response in the lung but provoked clear hematological alterations (increased numbers of circulating neutrophils and platelets, concomitant with a decline in circulating activated leucocytes) and clearly demarcating transcript expression changes in aorta pointing to an inflammatory systemic irritation after 24 h inhalation. In comparison, although many endpoints were addressed, intra-arterial infusion of CNP induced only a very modest systemic response. Thus, we conclude that indirect (local particle-cell interactions in the lung) rather than direct effects due to translocated CNP are mainly governing the observed systemic effects. Taking into account the results from our previous study on platelet accumulation in the hepatic microvasculature [36] using identical inhalation exposure settings (440 μg/m^3^, mass median diameter 72 nm), we suggest that the extra-pulmonary pro-thrombogenic effects represent the most sensitive response to CNP inhalation. These pro-thrombogenic effects may occur even in absence of any detectable pulmonary or systemic inflammation as previously reported [31, 35]. The lack of any signs for extra-pulmonary inflammation reported by the Khandoga et al., [35] study might be related to the use of C57BL/6 mice, which are less prone to inflammatory reactions than the BALB/c mice used here.

### Limitations of the study

For technical reasons two different types of CNP had to be used for inhalation (spark-discharge lab-generated CNP) and IAI (Printex90) exposure. While this may be considered as a weakness of the current study, there is compelling evidence that both CNP types are very similar in terms of organic carbon content, oxidative potency measured as ascorbic acid consumption (cell free) and induction of lipoperoxidation in macrophages, when expressed on a surface area basis [45]. Moreover, using BET surface area is used as dose metric both CNP types have been shown to display virtually identical pro-inflammatory potencies (summarized in Additional file 1: Table S8) suggesting similar underlying pathways of toxicity [28]. Intratracheal delivery of both CNPs types into mice, equally affected peripheral blood cell numbers and similarly as shown in Fig. 3. This compliant pattern for spark-discharge and Printex 90 material, further supports the comparability of the two CNPs. Nevertheless, the extra pulmonary effects of inhaled Printex90 or intra-arterially infused Palas particles remain hidden, and the use of different CNP materials remains a weak spot of the study which cannot be overlooked. To consider the resulting uncertainties we have aimed for a conservative dose adjustment and applied a maximal reasonable translocated fraction of inhaled CNP by IAI, so as not to underestimate the potential direct effects. Therefore, we argue that adjusting the IAI-delivered systemic CNP surface area dose to the upper limit of systemically available (translocated) CNPs after inhalation (30 mm^2^) allows a comparison of direct and indirect CNP effects at equivalent doses [41, 57]. Considering that on the one hand IAI-induced effects are smaller than those induced by inhaled CNPs, while on the other hand the CNP dose delivered via IAI is larger for a given period of time than the systemically available CNP dose after inhalation, we argue that the lack of response after IAI application cannot be seen as a result of low IAI dosing. Instead, we consider the lack of response after IAI suggests that particle-cell interactions in the lung (indirect effect), to represent the key event of the observed extra-pulmonary pro-thrombogenic effects following CNP inhalation. Another limiting factor could be that interactions of the Krebs-Henseleit buffer (IAI vehicle) with the suspended Printex90 particles may have altered the surface properties of the IAI delivered CNPs as compared to the inhaled-translocated CNPs. In 2% serum dispersed Printex90 CNPs, however can even enhance acute pulmonary inflammation upon instillation in comparison to pure water dispersions (unpublished observation). Nevertheless, the very same IAI application of these Printex CNPs was able to cause systemic effects in the hepatic microvasculature [34], arguing against persistent passivation of Printex90 particle by incubation in Krebs-Henseleit buffer. It is also possible that the higher stability of Printex90 in aqueous media to be due to hydrophilic surface properties (e,g, high abundance of oxygenated functionalities) which could likely be absent in the spark discharge generated CNPs that appear to be more hydrophobic. Moreover, a purity of 95–98% may correspond to significant differences of 2–5% impurity, is located particularly on the surface of particles. These differences in particle characteristics between Printex 90 and spark discharge generated may be considered as possible confounding factors.

Another potential drawback of the present study may be the influence of the surgical procedure required for IAI of CNPs which may have potential influence on the expression of protein markers and transcripts. Also, we focused the IAI- induced analysis on the 4 h time window because our previous studies indicated a 2 h interval to be sufficient for platelet accumulation in the hepatic microvasculature [34]. Pooling of samples for transcript and protein analysis may also induce false positives and negatives due to high inter sample variation in one group, such as extreme high or low values in one or two samples. To further reduce the impact of individual variations, we focused our analysis on changes observed for marker patterns, in order to achieve a more integral view. Additionally, though our present findings following 4 h IAI exhibited no substantial response, and also previous IAI results [34] do not demand for investigations at later time points, yet we consider that using additional animals to obtain IAI data at 24 h might have been ideal to exclude that later response have not been overlooked.

In spite of the limitations listed above, we are not aware of any other study that allows for similar types of conclusions and a direct comparison of dose-equivalent systemic effects after inhalation-translocation and IAI of CNPs. The methodological strength of the present study is that the intra-arterially infused CNP dose was chosen to match the estimated dose translocated from the lung epithelium into the circulation after inhalation, so that in the former case the particles circumvent pulmonary accumulation and possible release of inflammatory mediators from the parenchyma of the lung. Finally, it is noteworthy that our results are consistent with the human inhalation exposure study using identical spark-discharge generated CNP, which reported alterations of peripheral blood leukocyte distribution and expression of adhesion molecules consistent with increased leukocyte retention in the vascular bed [36]. However, in a real-life exposure scenario, ambient particulate matter will represent exposure a mixture of particles, gases, volatile organic compounds etc. This can only be mimicked by using collected ambient dust. Thus, the exposure scenario used in the current experimental set up is restricted to the toxicology of insoluble CNPs.

## Conclusion

This study shows that intra-arterial infusion of CNPs induced only mild early effects in secondary (non-pulmonary) target organs whereas inhalation of CNPs resulted in both pulmonary and extra-pulmonary (particularly in the aorta) responses. This indicates that the extra-pulmonary effects after CNP inhalation require local, pulmonary particle-cell interactions (indirect effect) and that the translocated CNPs (direct effect) appear to be of minor importance for the effects detected in extra-pulmonary targets. Interestingly, the molecular signature of the aorta proved to be most affected among the investigated extra-pulmonary tissues, displaying strong pro-inflammatory reactions coupled with increased levels of markers of atherosclerosis and endothelial dysfunction. However, these findings are only indicative of potential susceptibility that warrants detailed pathomechanistic investigations as vascular dysfunction has not been investigated here. Our data demonstrates that inhalation-mediated specific particle-lung cell interactions following CNP exposure might alter the systemic homeostasis leading to the retention of activated blood leucocytes in the pulmonary vascular bed and might induce a set of extra-pulmonary molecular reactions. Finally, the significant effect observed in the aortic tissue suggests the aorta to be an important extra-pulmonary target of CNP-mediated effects following the pulmonary response to inhaled CNPs that warrants more detailed investigations in future studies.

## Methods

### Animals

**Fig. 7:**
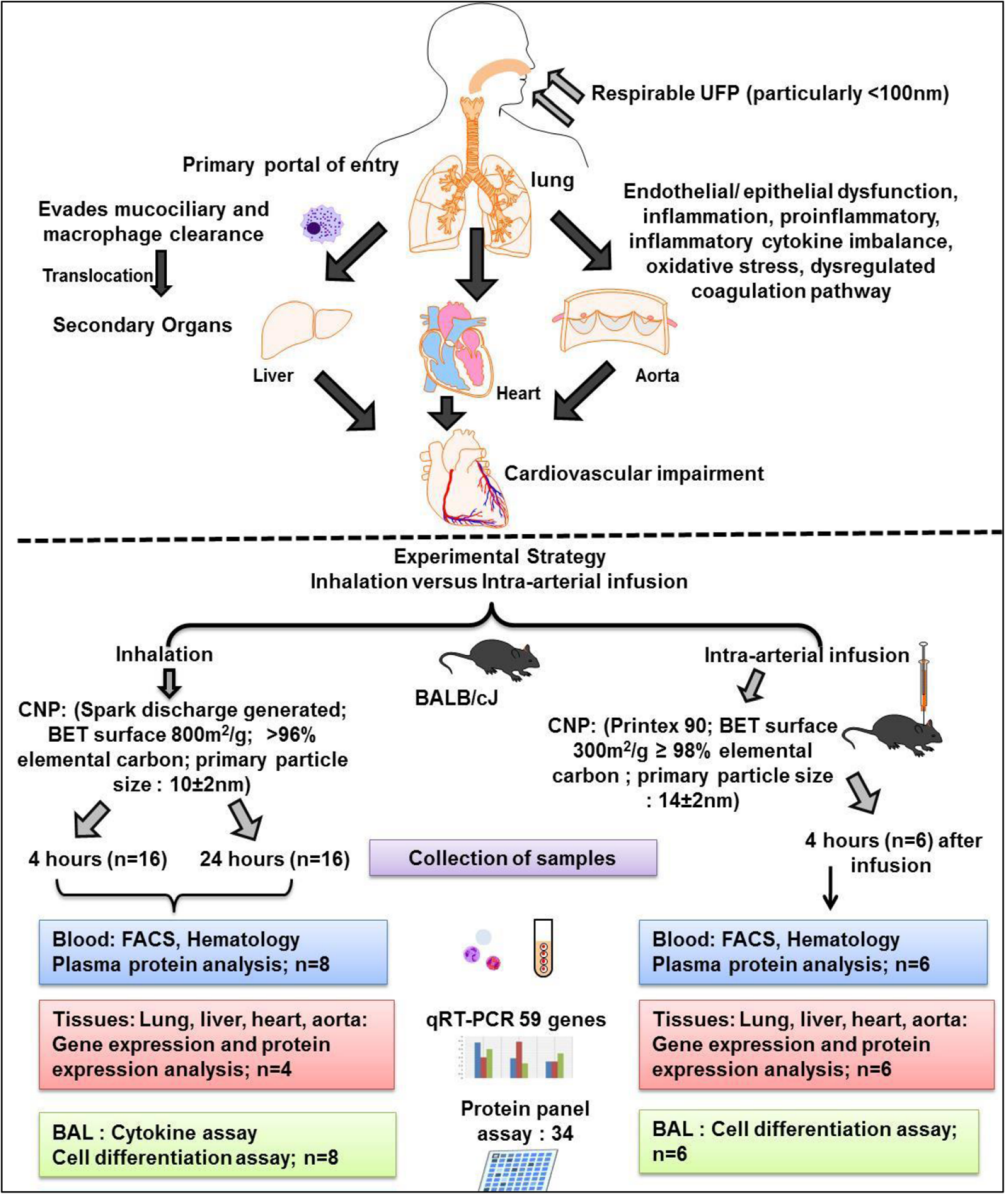
Schematic representation of the experimental strategy. The figure outlines the rationale and corresponding experimental strategy to compare and contrast the pulmonary and extra-pulmonary effects of carbon nanoparticle (CNP) exposure following inhalation and intra-arterial infusion in BALB/cJ mice. BAL: bronchoalveolar lavage; BET: Brunauer–Emmett–Teller; CNP: carbon nanoparticle; FACS: fluorescence automated cell sorting

Female BALB/cJ mice (10–12 weeks) housed under specific pathogen free conditions at the animal facility of the Helmholtz Zentrum München were used for the experiments. The inhalation and IAI groups consisted of *n* = 16 and *n* = 6 animals, respectively, for each of the experimental groups per time point. All experimental procedures were approved by the Bavarian Animal Research Authority (Approval no: 55.2–1–54-2531-115-05) in accordance with German law of animal protection. Animal numbers, experimental groups and overall experimental design are described in Additional file 1: Table S8 and Fig. 7.

### Particle types used for inhalation and intra-arterial infusion (IAI)

Ideally, identical CNPs should be used for inhalation and IAI exposure. For delivery of the highest possible lung-deposited CNP (surface area) dose rate in the ultrafine size range (<100 nm) via inhalation laboratory-generated spark-discharge CNP aerosol is the most suitable particle type [58]. Unfortunately, this particle type revealed to be not sufficiently stable in the Krebs-Henseleit buffer, the vehicle used for IAI application, and thus the previously used [34] commercially available carbon nanoparticles (Printex 90, Degussa, Frankfurt, Germany) were used for IAI exposure. These two types of particles have very similar physicochemical properties (spark discharge/Printex 90 CNPs), namely chemical composition (≥98%/≥95% elemental carbon, no bioavailable organic compounds), agglomerated spherical primary carbon particles with primary particle diameter of 10 ± 2 nm/14 ± 2 nm; Brunauer–Emmett–Teller (BET) surface area of 800 m^2^/g / 300 m^2^/g [24, 44, 45, 59]. Importantly, for both types of highly pure carbon particles the acute pulmonary toxicity in mice scales extremely well with BET surface area [44] and is directly related to the pro-oxidative surface activity, i.e., the surface specific toxicity is identical among the two types of CNPs [28]. These particle characteristics are summarized in the Additional file 1: Table S9. The preference of surface area as most predictive dose metric (rather than mass or number) for acute pulmonary effects, has not only been shown for a wide variety of carbonaceous but also for metal oxide and polymeric materials [60, 61]. Thus, BET surface area was used as dose metric to determine an equivalent dose of infused CNPs (Printex 90; mass-specific BET surface area: 300 m^2^/g) corresponding to the translocated dose of CNPs (spark discharge; 800 m^2^/g) after inhalation exposure [28, 44].

Inhalation of CNPs: The set-up of the whole-body exposure system for rodents used in this study has been previously described [30, 62]*.* Mice (*n* = 16) were simultaneously exposed for 4 or 24 h to either filtered air or spark discharge generated CNPs at a mass concentration of 440 μg/m^3^ corresponding to a BET surface area concentration of 3.5 × 10^5^ mm^2^/m^3^ with a number- and mass-based median diameter of 48 and 72 nm, respectively, and a geometric standard deviation (width) of the size distribution of 1.55. For these conditions, more than 80% of the CNP mass is in the ultrafine size range (below 100 nm). Please refer to Additional file 1 for more details.

### Translocated surface area dose after CNP inhalation

For assessment of the role of direct systemic particle effects after inhalation exposure, CNPs were intra-arterially infused (IAI) directly into the blood stream. The infused CNP surface area dose was chosen to match the CNP dose likely to be translocated from the lung to the blood stream during a 24 h inhalation exposure. As a worst case scenario the maximal translocated particle dose was estimated from the inhaled CNP surface area concentration (3.5 × 10^5^ mm^2^/m^3^) and literature values for the inhaled air volume during 24 inhalation (66 ml/min × 24 h = 0.095 m^3^) [63]; the lung deposited aerosol fraction of 34% for nanoparticles with a similar size distribution as used here [64] and the translocated fraction of the lung deposited dose of 0.3%, which implies that the lung deposited surface area dose is 330-fold higher (10,000 mm^2^) than the translocated dose. This number is derived from 0.1% translocation according to Kreyling et al., [23] and a safety factor of 3 accounting for uncertainties due to differences in particle type and animal model). As all of the relevant parameters were estimated conservatively (towards the highest possible dose, the resulting translocated dose of 30 mm^2^ for 24 h CNP inhalation can be considered an upper limit of the translocated CNP dose [Additional file 1, extended materials and methods].

### IAI of CNP

For IAI, commercially available CNPs (Printex 90, Degussa, Frankfurt, Germany), suspended in 200 μl Krebs-Henseleit buffer, were infused in mice (*n* = 6) through a catheter introduced into the aortic arch via the carotid artery and analyzed after 4 h. Mice intra-arterially infused with only Krebs-Henseleit buffer (i.e., vehicle) served as the corresponding control [34], [Additional file 1, extended materials and methods].

Similar to the inhalation route, more than 50% of the applied (agglomerated) CNPs mass was in the ultrafine size range (UfCP, mass-weighted (mobility) diameter below 100 nm). This was accomplished by carefully processing the initial CNPs suspension according to a sequence of filtration and vortexing steps [65]. For comparison of the biological response of inhaled-translocated and intra-arterially infused CNPs both doses were matched. Using the dosimetry method for CNPs described by Stampfl et al. [65] 30 mm^2^ of CNPs, which corresponds to 110 ng of CNPs (300 m^2^/g for Printex 90), were infused representing the maximum dose of inhaled-translocated CNPs as described above. Thus, the observed biological effects after intra-arterial infusion represents an upper limit of the expected direct effect of particle translocation from the lung to the blood stream after 24 h CNP inhalation (and even more so after 4 h inhalation).

### Animal procedures and molecular analysis

Mice were anesthetized by intraperitoneal injection of xylazine (4.1 μg/g) and ketamine (188.3 μg/g), blood was withdrawn from the retro-orbital plexus and collected in EDTA tubes (Sarstedt, Hannover, Germany) followed by exsanguination and bronchoalveolar lavage (BAL) as previously described [44]. Analysis of BAL cell differentials, BAL total protein content, hematology, and lung histology was performed. To abide by the “3R” (replace, reduce and refine) principles of animal protection, inflammatory response in the lungs of IAI exposed mice was assessed based on the most sensitive method in a minimal number of mice, namely expression analysis only (no BAL analysis), since only very low pulmonary effects were expected. Transcript and/or protein expression analysis for both control and experimental groups (4 h and 24 h inhalation, 4 h intra-arterial infusion) were performed using BAL fluid, plasma, lung, heart, liver, and aorta using panel assays as described in the supplementary section. Blood samples were also analyzed for monocyte and granulocyte activation using flow cytometry (LSR II, Becton Dickinson) and FlowJo Software (Version:7.2.2, Tree Star, Oregon). Granulocytes were defined as [GR1^+^Ly6G^+^] and monocytes as [GR1^+^Ly6G^−^] cells. Additionally, we also investigated three integrin cell surface markers, namely integrin alpha M (CD11b), alpha-4 integrin (CD49d) and beta-2 integrin (CD18) due to their established role in leukocyte-endothelial interaction and their association with human particle inhalation [36]. For transcript and protein expression analysis, samples were pooled from 4–6 animals for each experimental group.

### Statistical analysis

Statistical analysis was performed by student’s t-test comparing time-matched control and CNP exposed groups. Statistical significance was set at *p* < 0.05. Values are given as mean ± standard error of the mean (SEM). CNP exposure-related effects are typically expressed as changes relative to the time matched control.

## Additional file

Additional file 1: Table S1. Summary of the main findings following carbon nanoparticle (CNP) inhalation and intra-arterial infusion in BALB/cJ mice. ↑: Increased ↓: Decreased. **Table S2**: BAL cell differentials are shown in counts 4 and 24 h after clean air (Contol) or carbon nanoparticle (CNP) inhalation. Values measured out of range were defined as: <OOR. Macro: macrophages, Lympho: lymphocytes, Neutro: neutrophil granulocyte, EOS: eosinophil. Means ± SEM, Inhalation: *n* = 8; infusion: *n* = 6; * *p* ≤ 0.05. **Table S3**: BAL cytokine concentrations: Protein markers in BAL samples of mice 4 h and 24 h after clean air (Control) or carbon nanoparticle (CNP) inhalation. Values measured out of range were defined as: <OOR. CCL2: Monocyte chemoattractant protein-1 (MCP-1), CXCL1: Growth-regulated alpha protein (KC), G-CSF: Granulocyte colony stimulating factor, GM-CSF: Granulocyte-macrophage colony stimulating factor 2, IFN-gamma: Interferon gamma, IL: Interleukin, TNF-alpha: Tumor necrosis factor alpha. Mean ± SEM, * *p* ≤ 0.05. *n* = 8. **Table S4**: Gene annotation and primer sequences of the transcripts analyzed: List of the 59 transcripts analyzed by quantitative RT-PCR with respective primer pairs. Expression of Hprt1 expression served as control. **Table S5**: Haematological analysis Haematological analysis of blood samples 4 and 24 h after clean air (Control) or carbon nanoparticle (CNP) inhalation and 4 h after sham (Control) or CNP infusion. RBC: red blood cells, WBC: white blood cells, Lympho: lymphocytes, Neutro: neutrophil granulocyte, EOS: eosinophil granulocyte, Baso: basophil granulocyte, Mono: monocytes, PLT: platelets, Large PLT: large platelets. Means ± SEM, * *p* < 0.05. Inhalation: *n* = 8/group; Infusion: *n* = 6/group. **Table S6**: Multiplex protein suspension array Protein markers in plasma samples of mice 4 and 24 h after clean air (Control) or carbon nanoparticle (CNP) inhalation and 4 h after sham (Control) or CNP infusion. Values measured out of range were defined as: <OOR. CCL2: Monocyte chemoattractant protein-1 (MCP-1), CCL5: RANTES, CXCL1: Growth-regulated alpha protein (KC), E-selectin: Endothelial leukocyte adhesion molecule 1, G-CSF: Granulocyte colony stimulating factor, GM-CSF: Granulocyte-macrophage colony stimulating factor 2, IFN-gamma: Interferon gamma, IL: Interleukin, MMP9: Matrix metalloproteinase-9, sICAM-1: soluble Intercellular adhesion molecule 1, sVCAM-1: soluble Vascular adhesion molecule 1, TNF-alpha: Tumor necrosis factor alpha, total PAI-1: total Plasminogen activator inhibitor 1. Inhalation: *n* = 8; infusion: *n* = 6; Mean ± SEM, * *p* < 0,05. **Table S7**: Multiplex protein expression Protein expression analysis (pg/ml) of lung, heart and liver tissue 4 and 24 h after clean air (Control) or carbon nanoparticle (CNP) inhalation and 4 h after sham (Control) or CNP infusion. CCL2: Monocyte chemoattractant protein-1 (MCP-1), CCL3: MIP-1-alpha, CCL4: MIP-1-beta, CRP: C-reactive protein. CXCL1: Growth-regulated alpha protein (KC), CXCL2: Macrophage inflammatory protein 2 (MIP2), CXCL12: Stromal cell-derived factor 1 (SDF-1), E-selectin: Endothelial leukocyte adhesion molecule 1, GM-CSF: Granulocyte-macrophage colony stimulating factor 2, ICAM-1: Intercellular adhesion molecule 1, IFN-gamma: Interferon gamma, IL: Interleukin, L-selectin: Leukocyte adhesion molecule 1 (CD62L antigen), MMP2: Matrix metalloproteinase-2, MMP9: Matrix metalloproteinase-9, PDGF-AA: Platelet-derived growth factor A chain (dimer), PDGF-BB: Platelet-derived growth factor B chains (dimer), P-selectin: Leukocyte-endothelial cell adhesion molecule 3 (CD62P antigen), TGF-beta-1: Transforming growth factor beta-1, TNF-alpha: Tumor necrosis factor alpha, TNF-RI: Tumor necrosis factor receptor superfamily member 1A, VCAM-1: Vascular adhesion molecule 1, VEGF: Vascular endothelial growth factor. Samples were pooled from 4 animals/experimental group for gene and protein analysis. **Table S8**: Animal distribution for each experiment. CNP: Carbon nanoparticle; FACS: Fluorescence automated cell sorter. **Table S9**: Comparison of the properties form Printex 90 (IAI) and Palas spark-discharge (Inhalation) carbon nanoparticles (CNP). (DOCX 106 kb)

## Abbreviations

Alox5: Arachidonate 5-lipoxygenase
BAL: Bronchoalveolar lavage
BET: Method by Brunauer–Emmett–Teller for determining particle surface area by physical adsorption of gas molecules on a solid surface
CCL: CC-chemokine ligand
CD11b: Integrin alpha M
CD18: beta-2 integrin
CD49d: alpha-4 integrin
CNP: Carbon nanoparticles
CXCL: Chemokine (C-X-C motif) ligand
Emr1: Adhesion G protein-coupled receptor E1 (Adgre1: F4/80)
GM-CSF: Granulocyte-macrophage colony-stimulating factor
IAI: Intra-arterial infusion
IL: Interleukin
Lcn2: Lipocalin 2
Lgals3: Galectin 3
Ly6g: Lymphocyte antigen 6 complex: locus G
MPC: Mean platelet component
MPM: Mean platelet mas
MPV: Mean platelet volume
Ncf1, −2: Neutrophil cytosolic factor 1, −2
NP: Nanoparticles
Olr1: Oxidized low density lipoprotein
PAI-1: Plasminogen activator inhibitor-1
PCDW: Platelet component distribution width
PM: Particulate matter
PTC: Plateletcrit
Saa3: Serum amyloid A 3
SEM: Standard error of the mean
SHRs: Spontaneously hypertensive rats
Spp1: Secreted phosphoprotein 1 (Osteopontin)
UfCPs: Ultrafine carbon particles
