# Supplementary material for: Early pulmonary response is critical for extra-pulmonary carbon nanoparticle mediated effects: comparison of inhalation versus intra-arterial infusion exposures in mice
Source: Part Fibre Toxicol. 2017 Jun 20;14:19. doi: 10.1186/s12989-017-0200-x (PMC5480131; doi:10.1186/s12989-017-0200-x)
Supplement: Additional file 1: Table S1. — Summary of the main findings following carbon nanoparticle (CNP) inhalation and intra-arterial infusion in BALB/cJ mice. ↑: Increased ↓: Decreased. Table S2: BAL cell differentials are shown in counts 4 and 24 h after clean air (Contol) or carbon nanoparticle (CNP) inhalation. Values measured out of range were defined as: <OOR. Macro: macrophages, Lympho: lymphocytes, Neutro: neutrophil granulocyte, EOS: eosinophil. Means ± SEM, Inhalation: n = 8; infusion: n = 6; * p ≤ 0.05. Table S3: BAL cytokine concentrations: Protein markers in BAL samples of mice 4 h and 24 h after clean air (Control) or carbon nanoparticle (CNP) inhalation. Values measured out of range were defined as: <OOR. CCL2: Monocyte chemoattractant protein-1 (MCP-1), CXCL1: Growth-regulated alpha protein (KC), G-CSF: Granulocyte colony stimulating factor, GM-CSF: Granulocyte-macrophage colony stimulating factor 2, IFN-gamma: Interferon gamma, IL: Interleukin, TNF-alpha: Tumor necrosis factor alpha. Mean ± SEM, * p ≤ 0.05. n = 8. Table S4: Gene annotation and primer sequences of the transcripts analyzed: List of the 59 transcripts analyzed by quantitative RT-PCR with respective primer pairs. Expression of Hprt1 expression served as control. Table S5: Haematological analysis Haematological analysis of blood samples 4 and 24 h after clean air (Control) or carbon nanoparticle (CNP) inhalation and 4 h after sham (Control) or CNP infusion. RBC: red blood cells, WBC: white blood cells, Lympho: lymphocytes, Neutro: neutrophil granulocyte, EOS: eosinophil granulocyte, Baso: basophil granulocyte, Mono: monocytes, PLT: platelets, Large PLT: large platelets. Means ± SEM, * p < 0.05. Inhalation: n = 8/group; Infusion: n = 6/group. Table S6: Multiplex protein suspension array Protein markers in plasma samples of mice 4 and 24 h after clean air (Control) or carbon nanoparticle (CNP) inhalation and 4 h after sham (Control) or CNP infusion. Values measured out of range were defined as: <OOR. CCL2: Monocyte chemoattract [file 12989_2017_200_MOESM1_ESM.docx]

**Additional file 1**

**Early pulmonary response is critical for extra-pulmonary carbon nanoparticle mediated effects: Comparison of inhalation versus intra-arterial infusion exposures in mice**

**Short Title**: PM Lung interaction for systemic effects

Koustav Ganguly^1,2^, Dariusch Ettehadieh^3^, Swapna Upadhyay^1^, Shinji Takenaka^3^, Thure Adler^5^, Erwin Karg^3,6^, Fritz Krombach^7^, Wolfgang G. Kreyling^3^, Holger Schulz^8,9^, Otmar Schmid^3^, Tobias Stoeger^3^

**^1^**Unit of Lung and Airway Research, Institute of Environmental Medicine (IMM), Karolinska Institutet, SE-171 77 Stockholm, Sweden.

**^2^**Unit of Work Environment Toxicology, Institute of Environmental Medicine (IMM), Karolinska Institutet, SE-171 77 Stockholm, Sweden.

^3^Institute of Lung Biology and Disease, Comprehensive Pneumology Center, Helmholtz

Zentrum München, German Research Center for Environmental Health, Neuherberg,

Germany D85764

**^5^**German Mouse Clinic, Institute of Experimental Genetics, Helmholtz Zentrum München, German Research Center for Environmental Health, Neuherberg, Germany D85764

**^6^**Cooperationgroup Comprehensive Molecular Analytics (CMA), Joint Mass Spectrometry Centre (JMSC), Helmholtz Zentrum München, German Research Center for Environmental Health, Neuherberg, Germany D85764

**^7^**Walter Brendel Centre of Experimental Medicine, Ludwig-Maximilians-Universität München Germany D81377

**^8^**Institute of Epidemiology I, Helmholtz Zentrum München, German Research Center for

Environmental Health, Neuherberg, Germany D85764

^9^Comprehensive Pneumology Center Munich (CPC-M), Member of the German Center for Lung Research, Munich, Germany D85764

**Correspondance: Dr. Tobias Stoeger**

Institute of Lung Biology and Disease, Comprehensive Pneumology Center, Helmholtz Zentrum München, German Research Center for Environmental Health, Neuherberg,

Germany D85764

**Email:** tobias.stoeger@helmholtz-muenchen.de

**Phone:** +49-89-31873104; **Fax:**+49-89-31872400

**Extended Materials and methods section:**

**Animals:** Female BALB/cJ mice (Catalog #: 000651) were purchased from Jackson laboratory (Bar Harbor, ME, USA) and were housed in ‘isolated ventilated cages’ (IVC-Racks; BioZone, Ramsgate, United Kingdom) under specific pathogen-free (SPF) conditions according to the Federation of European Laboratory Animals Science Association (FELASA) guidelines at the animal facility of Helmholtz Zentrum München, German Research Center for Environmental Health. The mice were kept in filtered air and 12h light/12h dark cycle and were acclimatized for at least 2 weeks. Food and water were provided *ad libitum*. 10-12 weeks old mice were used for the experiments. The inhalation and intra-arterial infusion groups consisted of n=16 and n=6 animals respectively for each of control and exposure groups per time point. Animal distribution for each experiment is detailed in **Additional file 1: Table S8**.

**Particle types (CNP) and dose metric used for inhalation and intra-arterial infusion:** The methodological strength of the present study is that the intra-arterially infused CNP dose was chosen to match the estimated dose translocated from the lung epithelium into the circulation after inhalation, so that in the former case the particles circumvent pulmonary accumulation and possible release of inflammatory mediators from the parenchyma of the lung. Moreover, for both routes of application the majority of the CNP mass (here: >60%) was in the ultrafine size range (<100 nm in diameter). In order to meet these requirements, two similar, but slightly different types of CNPs had to be used.

While inhalation of aerosolized CNPs at high mass concentrations and yet small particle diameter – as required here - can only be performed with in-situ generated CNPs, infusion is only possible for CNPs, which are stable in Krebs-Henseleit buffer, the vehicle used for particle infusion. Since there is no CNP type which is meeting both sets of requirements, two different but physicochemically very similar types of CNPs were used here, namely freshly produced spark-discharge CNP aerosol and commercially available CNPs (Printex 90, Degussa, Frankfurt, Germany). The criteria for choosing those two types of CNPs are the physicochemical similarity (spark discharge/Printex 90 CNPs: chemical composition: ≥98%/≥95% elemental carbon, no bioavailable organic compounds; agglomerated spherical primary particles with primary particle diameter of 10±2/14±2nm; (Frampton 2004; Stoeger et al., 2006, Matuschek et al., 2007 and Stoeger et al., 2009)) and the fact that their acute pulmonary toxicity in mice scales with BET surface area (surface area determined according to the method described by Brunauer, Emmett and Teller), i.e., their surface specific toxicity is independent of particle type (Stoeger et al., 2009). Thus, BET surface area can be used as dose metric to determine an equivalent dose of infused CNPs (Printex 90; mass-specific BET surface area: 300 m^2^/g) corresponding to the translocated dose of CNPs (spark discharge; 800 m^2^/g) after inhalation exposure (Stoeger et al., 2006, Stoeger et al., 2009). It is noteworthy that the spark discharge CNPs have also been used for inhalation studies with healthy and diseased volunteers (Frampton et al., 2004; Frampton et al., 2006; Schaumann et al., 2014; and Vora et al., 2014).

**Inhalation of CNP:** The set-up of the whole-body exposure system for rodents used here has been described previously by Karg et al., (1998) and Andre et al., (2006)***.*** Briefly, the exposure chamber was supplied with a constant flow of aerosol-laden humidified air (23°C, 46% relative humidity). CNP aerosol was produced by an improved electric spark discharge generator (Model GFG 1000; Palas, Karlsruhe, Germany) operated with ultrapure graphite electrodes in an argon atmosphere (<10^-6^ impurities) (Roth et al., 2004) and modified to avoid inadvertent outgassing of organic compounds by replacement of all wetted parts with materials free of organics. Mice (n = 16) were exposed for 4 or 24h to either filtered air or spark discharge generated CNPs. The mean number and mass concentration of the aerosolized CNPs was 6.4 x 10^6^ cm^‑3^ and 440 µg/m^3^, respectively, with a number- and mass-based median diameter of 48 and 72 nm, respectively, as determined by gravimetric analysis and scanning mobility particle sizing (EMS 150; Hauke, Gmunden, Austria; CPC 3022A; TSI, St. Paul, MN, USA). The conversion of number size distribution into mass distribution was performed taking into account that the equivalent density of fractal-like carbon (soot) particles changes is indirectly proportional to the mobility diameter (~d^-1^) (Gwaze et al., 2006). For a typical geometric standard deviation (width) of the CNP aerosol size distribution of 1.55 about 80% of the particle mass was in the size regime below 100 nm (ultrafine size range). Finally, conversion of mass into BET surface area using the mass-specific BET surface area of spark discharger CNPs (800 m^2^/g) yielded CNP surface area concentration of 3.5 x 10^5^ mm^2^/m^3^.

**Intra-arterial infusion (IAI) of CNP:** For intra-arterial infusion, commercially available CNPs (Printex 90, Degussa, Frankfurt, Germany) were suspended in Krebs-Henseleit buffer, since – as stated above - spark-discharge generated CNPs (as used for inhalation) were not sufficiently stable in suspension for IAI. Subsequently, a known surface area dose of CNPs (30 mm^2^; see below) was infused in mice (n = 6) through the aortic arch via carotid artery. Mice intra-arterially infused with only Krebs-Henseleit buffer (i.e. vehicle) served as the corresponding control (Khandoga et al., 2004). As previously reported by us (Khandoga et al. 2004), pro-thrombotic effects in the hepatic microcirculation were detected already 2h after intra-arterial infusion of 5 x 10^7^ CNPs with approximately 60% of particles in the suspension having a diameter of <100nm (see below). Therefore, we assigned the time point of investigation at 4h following intra-arterial infusion to detect CNP triggered transcriptional activation and protein biosynthesis.

For Printex 90 CNPs ultrasonication and vortexing alone was insufficient to obtain high enough CNP concentrations in the ultrafine size range. Thus, a sequence of vortexing and filtration steps (filter pore size 100 nm) was performed to provide a stable particle suspension with at least 60% of the particle mass in the size range below 100 nm as determined by dynamic light scattering (Zetasizer, Malvern; Stampfl et al., 2011). The filtration steps made it impossible to infer the CNP mass concentration from the gravimetrically determined invested CNP mass. Hence, a method introduced by Stampfl et al., 2011 was used to determine the number concentration and the associated surface area concentration of CNPs (agglomerates) in suspension from the intensity data obtained by dynamic light scattering (DLS) analysis of the suspension. In brief, using polystyrene latex (PSL) spheres of defined size and number concentration it was revealed that the observed DLS light intensity peaks of 40 nm and 100 nm PSL particles scaled with their diameter (~d^5.4^, which is close to the expected Rayleigh limit of ~d^6^, Stampfl et al. 2011). The number of CNP agglomerates having an average diameter of 100 nm can then be calculated by titration of known numbers of 40 nm PSL particles into an aliquot of the CNP suspension to be infused until the dynamic light scattering intensities of the observed PSL and CNP peaks (Zetasizer) were equal, yielding the number concentration of CNPs as N_CNP_ = N_PSL_ *(40 nm/100 nm)^5.4^. Using this method, the translocation-matched CNP surface area dose of ca. 30 mm^2^ required IAI of 5 x 10^7^ CNP agglomerates (for details see next section).

Similarities and differences in the physocichemical properties as well as biological responses are summarized in Additional file 1: Table S9. A column giving the respective characterization data of the Ptx90 CNP as % from that of the Palas CNP has ben added to display the level of similarity between the two different CNPs.

**Matching translocated and infused surface area dose:**  For dosimetrically equivalent comparison of the biological response induced by translocated (after inhalation) or directly infused CNPs, the intra-arterially applied CNP surface area dose was chosen to match the systemically translocated particle dose following the inhalation exposure. First, the upper limit of the translocated particle dose for 24h inhalation was estimated based on the measured aerosol characteristics in the inhaled air (mass concentration, size distribution). Then the translocated dose was determined using available data from the literature on inhaled air volume, particle lung deposition and the translocated particle fraction of BALB/c mice. If a range of values for a given parameter is available, those values maximizing the estimated translocated dose were chosen to provide guidance on the “worst case” scenario to be matched by intra-arterial infusion. In brief, the inhaled aerosol mass (42 µg) was calculated from the measured aerosol mass concentration (440 µg/cm^3^) in the inhaled air and the inhaled volume of air (BALB/c mice: tidal volume: 0.2ml, breathing rate: 330/min for 24h inhalation: 9.5 m^3^ (DeLorme and Moss, 2002); inhalation time period: 24h). About 34% of the inhaled mass of spark-discharge generated iridium nanoparticles with a similar particle morphology and size distribution as obtained here (count median diameter and geometric standard deviation was 35 nm and 1.7, respectively), was deposited onto the lung epithelium BALB/c mice (Alessandrini et al.. 2008) and up to 0.3% of that was estimated to be translocated via the air-blood barrier of the lung. The translocation efficiency of 0.3% was determined from a comprehensive translocation study with gold nanoparticles between 1.4 and 200 nm in rats (Kreyling et al., 2014) reporting less than 0.1% translocation for particles larger than 18 nm. An additional safety factor of 3 was included to account for possible uncertainties due to differences in particle type (carbon versus gold) and animal model (mouse versus rat). The resulting translocated mass of 42 ng corresponds to a surface area dose of 33 mm^2^ (mass-specific BET surface area: 800 m^2^/g; Stoeger et al., 2006).

The corresponding surface area dose of 33 mm^2^ for IAI corresponds to 110 ng of Printex 90 CNPs (using 300m^2^/g). For reasons mentioned above not the mass, but the number concentration of CNPs in the IAI vehicle was determined by dynamic light scattering analysis (Stampfl et al., 2011). For conversion of the targeted mass (surface area) dose into the corresponding number dose the scaling laws for fractal-like agglomerates were used. Utilizing the known diameter and density of the spherical primary particles of Printex 90 (*d_p_* = 14 nm; density of soot is 2 g/cm^3^), the mass of the primary particle was determined (2.9x10^-18^g). Moreover, the number of primary CNPs per 100 nm CNP agglomerate (average diameter) was 655 (primary particles) as derived from a well-known fractal scaling law, *N_p_*=k_f_*(d_agg_/d_p_)^Df^, where *D_f_* is the fractal dimension of the agglomerate (here estimated as 2.5), *d_agg_* is the mobility diameter of the CNP agglomerates (100 nm), *d_p_* is the diameter of the primary carbon particle (14 nm) and k_f_=4.8 (=2.77/0.8^Df^) (Gwaze et al., 2006, Baron and Willeke, 2001). Hence, the mass of a 100 nm CNP agglomerate is 1.9*10^-15^g yielding an equivalent targeted number dose of 5.8 x 10^7^ (=110 ng/1.9x10^-15^g). This applied number dose of 5 x 10^7^ matched the targeted value within experimental uncertainties.

In summary, we determined an estimated upper limit of the translocated particle surface area dose (ca. 30 mm^2^) after 24h inhalation and intra-arterially infused this CNP surface area dose directly into the blood stream allowing for assessment of direct CNP effects. The fact that the translocated dose was estimated as upper limit takes into account that the delivery of a CNP bolus during IAI may induce an enhanced response compared to the slow and persistent translocation rate during inhalation exposure.

**Mouse procedures:** Mice were anesthetized by intraperitoneal injection of xylazine (4.1 µg/g) and ketamine (188.3µg/g), blood was withdrawn from retroorbital plexus and collected in EDTA tubes (Sarstedt, Hannover, Germany) following which they were sacrificed by exsanguination. Bronchoalveolar lavage (BAL) was performed on control and CNP-inhaled and intra-arterially infused experimental groups as previously described (Stoeger et al., 2006). Briefly, BAL was performed by cannulating the trachea and infusing the lungs 10 times with 1.0 ml PBS without calcium and magnesium. The BAL fluid from lavages 1 and 2 and from lavages 3-10 were pooled and centrifuged (425 *g*, 20 min at room temperature). The cell-free supernatant from lavages 1 and 2 were pooled and used for biochemical measurements such as total protein and panel assays. One portion of the cell pellet was resuspended in 1 ml RPMI 1640 medium (Bio-Chrome, Berlin, Germany) and supplemented with 10% fetal calf serum (Seromed, Berlin, Germany); the number of living cells was determined by the trypan blue exclusion method. We performed cell differentials on the cytocentrifuge preparations (May-Grünwald-Giemsa staining; 2 times 200 cells counted). We used the number of macrophages and polymorphonuclear leukocytes (PMNs) or neutrophils as cellular markers of inflammation. The other portion of the cell pellet was stored at -80°C for molecular analysis. Total protein content was determined spectrophotometrically at 620 nm, applying the Bio-Rad Protein Assay Dye Reagent (catalog# 500-0006; Biorad, Munich, Germany). We analyzed 50 μl BAL/mouse to assess each panel assays. Hematological analysis was performed within 1h of blood collection using ADVIA 120 hematology system (Bayer, Fernwald, Germany). Briefly, non-lavaged, inhalation-exposed animals were used for histological analysis. To excise lungs, heart, liver, and aortic tissue for protein and transcript analysis, the diaphragm was punctured and the chest cavity opened in exsanguinated animals: Organs were collected and shock-frozen in liquid nitrogen and stored (-80ºC). Prior to collection of tissues, blood was collected from the abdominal aorta for all animals. Plasma was separated immediately as per standard procedures. Blood samples collected from each animal were stored in aliquots of 2.6 ml in 2.9 ml *S-Monovette*® tube (Sarstedt, Germany) with EDTA for further analysis of different markers. Each blood sample with anticoagulant was centrifuged (at 2710 g) for 10-minutes (4°C) for the collection of plasma sample or centrifuged for 15-minutes (at 1300 g, 4°C) and stored at -80°C until analyzed. Transcript and/ or protein expression analyses for both control and experimental groups (4h and 24h inhalation, 4h intra-arterial infusion) were performed using lung, heart, liver, and aorta. Analysis of the aorta was restricted to transcript level due to the less availability of tissue. All experimental procedures were approved by Bavarian Animal Research Authority (Approval no: 55.2-1-54-2531-115-05) and were in accordance with German law of animal protection.

**Flow cytometric analysis of leukocytes:** Blood samples were analyzed for monocyte and granulocyte activation using a fluorescence-activated cell sorter (FACS; LSR II, Becton Dickinson) and FlowJo Software (Version: 7.2.2, Tree Star, Oregon). Granulocytes were defined as [GR1^+^Ly6G^+^] and monocytes as [GR1^+^Ly6G^-^] cells according to manufacturer’s recommendation. Additionally, we also investigated three integrin cell surface markers, namely integrin alpha M (CD11b), alpha-4 integrin (CD49d) and beta-2 integrin (CD18) due to their established role in leukocyte-endothelial interaction and their association with human particle inhalation (Frampton et al., 2006).

**Protein homogenate preparation from tissues:** Protein extracts of whole organs were prepared for lung, heart and liver. Tissues were weighed and added to 9X volume of lysis buffer and homogenized. Total tissue homogenate was prepared using 50 mM Tris-HCL with 2 mM EDTA, pH 7.4 as the lysis buffer supplemented with a protease inhibitor cocktail (P8340 Sigma-Aldrich). Following homogenization the tissue preparation was centrifuged for 2 min in a microfuge at 13000 rpm (Eppendorf 5415R). Without disturbing the cell pellet the supernatant was aspirated. The total protein concentration in the homogenates was determined as described above.

**BAL cytokine analysis** was performed using BioPlex Mouse Cytokine Array (14 cytokines; Mouse 14-Plex, Biorad laboratories, Germany). Concentrations of cytokines were measured from the BAL fluid from n=8 animals/experimental group of inhalation exposure. Measurements were carried out using Luminex 100 device (Bio-Rad laboratories; Germany) and BioplexManager Software (Bio-Rad laboratories; Germany). The cytokines assayed were CCL2, CXCL1, GCSF, GMCSF, IFNγ, IL1α, IL1β, IL2, IL6, IL10, IL12(p40), IL13, IL17, and TNFα. Five (GMCSF, IL1β, IL10, IL17, and TNFα) out of 14 cytokines were below detectable range **(**Additional file 1: Table S2**).**

**Plasma protein analysis** consisted of 25 analytes in total using BioPlex Mouse Cytokine Array (14 cytokines; Mouse 14-Plex: product range have changed, Biorad laboratories, Germany) and Lincoplex cardiovascular disease (CVD) panels: CVDI and CVDII (11analytes; Millipore). Analysis was carried out using n=6-8 animals/experimental group for the multi analyte protein assays. Measurements were carried out using Luminex 100 device (Bio-Rad laboratories; Germany) and Bioplex Manager Software (Bio-Rad laboratories; Germany). 20 out of 25 proteins were in detectable range that included ADIPOQ, CCL2, CXCL1, fibrinogen, GCSF, GMCSF, IFNγ, IL1α, IL1β, IL2, IL6, IL10, IL12(P40), IL13, IL17, MMP9, sICAM1, sVCAM1, TNFα, total PAI-1 **(**Additional file 1: Table S3**)**.

**Lung, heart and liver protein analysis** have been performed using SearchLight® Proteome custom array (34 analytes; catalog #:SL4600; Thermofischer Scientific). The panel consisted of markers for endothelial/epithelial activation [ICAM1, SELP, SELE, VCAM1 and VEGF]; inflammation[CRP, MMP2, MMP9, SPP1, SELL, TNFR1]; and cytokines [ADIPOQ, CCL2, CCL3, CCL4, CXCL1, CXCL2, CXCL12, GMCSF, IFNγ, IL1α, IL1β, IL2, IL6, IL12(p40), IL13, IL17, IL10, IL1Ra, PDGF-AA, PDGF-BB, RETN, TGFβ1, TNFa]. Protein homogenates were pooled from n=4 animal/inhalation experimental group and n=6 animal/intra-arterial infusion experimental group. Data are represented as fold change relative to control. Samples were pooled from 4 animals/experimental group for protein analysis.

**Transcript analysis:** Total RNA was isolated from lung, heart, liver and aorta using *QIAGEN RNeasy Mini Kit* (Qiagen, Germany) according to manufacturer’s instruction, quality confirmed by denaturing formaldehyde/agarose/SYBR Gold gel (Agilent Bioanalyzer Analysis), and quantified (PeqLab Nanodrop Bioanalyzer). cDNA was prepared from 1µg total RNA using Invitrogen superscript II kit according to manufacturer’s instruction. Quantitative real time polymerase chain reaction (qRT PCR) was performed as described previously (Andre et al., 2006) using the ABI Prism 7000 Sequence Detection System (Applied Biosystems). Target transcript expressions were normalized to *Hprt.* Primer pair sequences of 59 transcripts assayed are provided in Additional file 1: Table S1. Data are represented as fold change relative to control. Samples were pooled from 4 animals/experimental group for transcript analysis.

**Statistical analysis:** Statistical analysis was performed by student’s t-test comparing time-matched control and CNP exposed groups. Statistical significance was set at p≤0.05.Values are given as mean ± standard error of the mean (SEM). CNP exposure related effects are typically expressed as changes relative to the time matched control.

**Extended results section:**

**BAL fluid analysis:** A mild influx of macrophages was detected after 4h of CNP inhalation exposure (control: 5.5±0.1×10^5^; exposed-4h: 6.7±0.1×10E5 cells; p≤0.05; n=8) which was not observed after 24h. In contrast to an initial drop at 4h, a sharp granulocyte/neutrophil influx was noted in the BAL fluid of the exposed group at 24h post inhalation (control: 2.5±0.8×10E3; exposed-24h: 10.2±2.5×10E3 cells; p≤0.05; n=8). Decreased lymphocyte levels were detected at both 4h and 24h post inhalation (**4h:** control: 10.8±0.8×10E3; exposed-4h: 9.2±0.7×10E3 cells and **24h:** control: 15.4±0.9×10E3; exposed-24h: 8.2±0.9×10E3 cells; p≤0.05; n=8) [Additional file 1: Table S2]. Total protein levels in the BAL fluid was increased at 4h (control: 95.9±8.4; exposed-4h: 136.3±6.2 µg/ml) and reached to basal level at 24h [Additional file 1: Table S3]. Analysis of 14 cytokines in the BAL revealed decreased levels of CXCL1 (2.6 fold); IL1α (1.8 fold) and IL12(p40) (1.4 fold) in exposed animals at 4h following CNP inhalation exposure compared to control. At 24h post CNP inhalation, CXCL1 increased by 4.5 fold, in agreement with BAL polymorphonuclear neutrophils (PMN) numbers, whereas CCL2 and IL1α decreased by ~10 fold and 1.5 fold respectively in the exposed animals compared to control [Additional file 1: Table S3].

**Lung Tissue Analysis:** Transcripts of 59 genes [Additional file 1: Table S4] have been assayed in the lung tissue for their expression levels to characterize the local response after inhalation of CNPs. At 4h post inhalation, 32 genes were down-regulated by ≥1.5 fold and none were up-regulated compared to the control. The list includes *Fga* (>5 fold); *Fgg, Ly6g, S100a8* (3-4.9 folds); *Alox5, Cd68, Ifng, Il1b* (2-2.9 folds); *Cd40, Cd40lg, Sele, Thbs1, Vcam1, Ldlr, Lgals3, Lta4h, Mmp9, Ncf2, Nfkbib, Nos2, Olr1, Ptgs1, Spp1, Ccl2, Cxcl5, Il1a, Gclc, Gsta1, Hmox1,Csf2,Tnf*and *Sod3* (1.5-1.9 folds). At 24h post inhalation, 10 genes were up-regulated by ≥ 1.5 fold and none were down-regulated compared to the control. The up-regulated genes include: *Cxcl5* (>5 fold); *Fga, Cxcl1, Cxcl2* (3-4.9 folds); *Fgb, Fgg, Il10* (2-2.9 folds); and *Thbs1, Lcn2, Csf2* (1.5-1.9 folds).

Analysis of the 34 analytes covering markers for epithelial/endothelial activation and inflammation revealed 24 up-regulated (≥1.5 fold) and 1 down-regulated (≥5 fold) protein at 4h post inhalation whereas at 24h post inhalation 12 proteins were up-regulated (≥1.5 fold) and none down-regulated compared to their control**.** At 4h the up-regulated proteins are: TNFα (>5 fold); ICAM1, MMP2, CXCL12, GM-CSF, IL-10 (3-4.9 folds); VEGF, TNF-R1, IL-1β, IL-6, IL-17, PDGF-BB, RETN (2-2.9 folds); SELP, VCAM-1, SPP1, CCL3, CCL4, IFN-γ, IL-1α,IL-2, IL-12(p40), 1L-13 and IL-1Ra (1.5-1.9 folds). TGFβ1 is down-regulated by >5 folds at 4h post inhalation. The up-regulated proteins at 24 h post inhalation are MMP2, GM-CSF, IL-6, IL-10 (2-2.9 folds); and VEGF, CCL4, IL-1β, IL-2, IL-13, IL-17, RETN, TNF-α (1.5-1.9 folds).

Decreased transcript levels of *Fga, Fgb, Fgg* (>5fold) and *Ccl2* (1.5 fold) were detected in the lung 4h post intra-arterial infusion of CNP compared to control. 1.5-1.9 folds reduced TNF-R1, CCL2 and RETN protein levels were detected in the lung following intra-arterial infusion of CNP compared to control**.** Samples were pooled from 4 animals/experimental group for gene and protein analysis.

**Transcript and protein analysis in extra-pulmonary organs:**

Transcript and protein panel assays were carried out in heart, liver and aorta (Additional file 1: Table S7) to assess the effect of CNP exposure via inhalation and intra-arterial infusion in extra pulmonary organs. Samples were pooled from 4 animals/experimental group for gene and protein analysis.

**Heart Tissue Analysis:** 29 genes were differentially expressed (12 up-regulated and 17down-regulated) at 4h post inhalation in the heart tissue compared to control. The up-regulated genes included *Ly6g* (>5 fold); *Lgals3, Olr1, Spp1* (2-2.9 folds); and (F3, Fn1, *Lcn2, Csf2, Cxcl1, IFNg, Il1a, Il6* (1.5-1.9 fold). Genes that were down-regulated included *Cd40lg, Fgg, S100a8* (3-4.9 folds); *Fga, Fgb, Ldlr, Lta4h, Nfkbib* (2-2.9 fold); and *Alox5, Cd68, Emr1, Ncf2, Saa3, Ccl2, Tgfb1, Tnf, Sod3* (1.5-1.9 folds).

A total of **25 genes** were differentially regulated (11 up-regulated and 14down-regulated) at 24h post inhalation in the heart tissue compared to control. The up-regulated genes consist of *Ly6g, Cxcl1* (3-4.9 folds); *Lcn2, Il1a, Il6* (2-2.9 folds); and *Edn1, F3, Lgals3, Csf2, Cxcl2, Il10* (1.5-1.9 folds). The down-regulated genes consists of *Fgg* (>5 fold); *Ldlr* (3-4.9 folds); *Cd40lg, Fgb, Cd68, S100a8, Sod3* (2-2.9 folds); and *Fga, Alox5, Emr1, Ncf2, Pecam1, Saa3, Tgfb1* (1.5-1.9 folds).

Expressions of six proteins were altered at 4h post inhalation in the heart compared to control. SPP1 was induced by 2.5 fold, CXCL2 was induced by 1.5 fold, whereas SELP, CRP, IL-1α and IL-2 were decreased by 1.5-1.9 folds. At 24h post inhalation, 12 proteins were detected with altered expression (9 up-regulated and 3 down-regulated) compared to control. The up-regulated proteins consisted of IL13 (2-2.9 fold); CCL4, CXCL1, GMCSF, IL-6, IL-1RA, IL-12 (p40), IL-17, IL-10 (1.5-1.9 folds). The down-regulated proteins included SELP, TGF- beta-1 (3-4.9 folds); and RETN (1.5 – 1.9fold).

10 genes were differentially regulated (≥1.5 fold) in the heart at 4h following intra-arterial infusion of CNP compared to control. Transcripts of 3 genes, *Gsta1* (2.5 fold), *Spp1* (1.5 – 1.9 fold) and *Hmox1* (1.5–1.9 fold) were up-regulated. Seven genes namely, *Fgb, Fgg* (>5 folds); *Fga* (4 fold); *Olr1, Cxcl5, Il1a* and *Il6* (1.5-1.9 folds) were down-regulated. 1.5-1.9 folds reduced SELP and TGFβ-1 protein levels were detected in the heart following intra-arterial infusion of CNP compared to control.

**Liver Tissue Analysis:** 14 genes have been differentially expressed in the liver following 4h post inhalation compared to control of which only one is up-regulated (*Cxcl5*; 1.5 fold). The 13 down-regulated genes included: *Fn1, Alox5, Nos2* (3-4.9 folds); *Olr1, Ptgs1* (2-2.9 folds); and *Ace, F3, Thbs1, Vwf, Itgam, S100a8, Il10, Tnf* (1.5-1.9 folds).

At 24h post inhalation, 3 up-regulated and 13 down-regulated, totaling 16 differentially expressed genes were detected in the liver compared to control. Transcripts of *Fn1* (3 fold); *Il1a* (2.5 fold); and *Thbs1* (1.5 fold) were increased. *Olr1, Ccl2* (3-4.9 folds); *Cd40, Alox5, Lta4h, Nos2, Tgfb1* (2-2.9 folds); and *Cd40lg, F3, Ptgs1, S100a8, Csf2, Cxcl1* (1.5-1.9 folds) were decreased.

Altered levels of 18 analytes have been detected at 4h following CNP inhalation. The increased proteins were VEGF, IL1α, IL2, RETN (2-2.9 folds); and SELE, SELP, VCAM1, CRP, CXCL12, IFN-γ, IL-1β, IL12 (p40), PDGF-AA, PDGF-BB, TNFα, Osteopontin, TNFR1 and MMP2 [1.5-1.9 folds]. 5 differentially regulated analytes were detected in the liver at 24h post inhalation of CNP compared to control. TGFβ was increased >5 fold whereas SELL (2.5 fold), IL1α, IL2, TNFα (1.5-1.9 fold) were decreased.

17 genes were down-regulated in the liver at 4h following intra-arterial infusion of CNP compared to control. The list consists of *Edn1, Il6, Alox5, Ccl2,* and *Cxcl5* (2-2.9 folds); *Cd40, F3, Sele, Selp, Thbs1, Vcam1, Ly6g, Pecam1, Il10, Il1a, Il1b,* and *Tnf* (1.5-1.9 fold).2-2.9 fold increased SELL and TNFR1 protein levels were detected in the liver at 4h following intra-arterial infusion of CNP compared to control.

**Animals:** Female BALB/cJ mice (Catalog #: 000651) were purchased from Jackson laboratory (Bar Harbor, ME, USA) and were housed in ‘isolated ventilated cages’ (IVC-Racks; BioZone, Ramsgate, United Kingdom) under specific pathogen-free (SPF) conditions according to the Federation of European Laboratory Animals Science Association (FELASA) guidelines at the animal facility of Helmholtz Zentrum München, German Research Center for Environmental Health. The mice were kept in filtered air and 12h light/12h dark cycle and were acclimatized for at least 2 weeks. Food and water were provided *ad libitum*. 10-12 weeks old mice were used for the experiments. The inhalation and intra-arterial infusion groups consisted of n=16 and n=6 animals respectively for each of control and exposure groups per time point. Animal distribution for each experiment is detailed in Additional file 1: Table S8.

**Table S1:** Summary of the main findings following carbon nanoparticle (CNP) inhalation and intra-arterial infusion in BALB/cJ mice. ↑: Increased ↓: Decreased

| **ANALYSIS** | **INHALATION** | | | | **INTRA-ARTERIAL INFUSION** | |
| --- | --- | --- | --- | --- | --- | --- |
|  | **4h** | | **24h** | | **4h** | |
| **BAL CELL DIFFERENTIALS** | ↑macrophages; ↓neutrophil; ↓lymphocytes  ↑Total protein | | ↑neutrophil; ↓lymphocytes | | Not done | |
| **HEMATOLOGY** | ↑platelets; ↑large platelets; ↑Mean platelet volume  ↑Mean platelet mass; ↑platelet distribution width  ↑Plateletcrit; ↑Platelet component distribution  ↓mean platelet component | | ↑granulocytes; ↑monocytes; ↑platelets; ↑large platelets  ↑Mean platelet volume; ↑Mean platelet mass  ↑Plateletcrit; ↑Platelet component distribution  ↓mean platelet component | | ↑lymphocytes | |
| **FACS-BLOOD** | ↓CD18-Granulocyte; ↓CD18- Monocytes | | ↓CD49b-Granulocyte; ↓CD11b-Granulocyte  ↓CD18-Granulocyte; ↓CD49b-Monocytes  ↓CD18- Monocytes | | No change | |
|  | **UP REGULATED** | **DOWN REGULATED** | **UP REGULATED** | **DOWN REGULATED** | **UP REGULATED** | **DOWN REGULATED** |
| **BAL- PROTEIN**  **(≥ 2FOLD)** | none | CXCL1 (2.6X); | CXCL1(4.5X) | CCL2 (10.2X) | NOT DONE | NOT DONE |
| **LUNG –GENE (≥ 2FOLD)** | none | *Fga*, *Fgg, Ly6g, S100a8*, *Alox5, Cd68, Ifng, Il1b* | *Cxcl5,Fga, Cxcl1, Cxcl2*, *Fgb, Fgg, Il10* | none | none | *Fga, Fgb, Fgg* |
| **LUNG-PROTEIN (≥ 2FOLD)** | TNFα, ICAM1, MMP2, CXCL12, GM-CSF, IL-10, VEGF, TNF-R1,  IL-1β, IL-6, IL-17, PDGF-BB, RETN | TGFβ1 | MMP2, GM-CSF, IL-6, IL-10 | none | none | none |
| **Plasma protein (≥ 2FOLD)** | none | IL1α | none | GMCSF, IL1α | none | none |
| **HEART-GENE (≥ 2FOLD)** | *Ly6g*, *Lgals3, Olr1, Spp1* | *Cd40lg, Fgg, S100a8*, *Fga, Fgb, Ldlr, Lta4h, Nfkbib* | *Ly6g, Cxcl1Lcn2, Il1a, Il6* | *Fgg*, *Ldlr, Cd40lg, Fgb, Cd68, S100a8, Sod3* | *Gsta1* | *Fgb, Fgg* ,*Fga* |
| **HEART-PROTEIN (≥ 2FOLD)** | SPP1, | SELP | IL13, | SELP, TGFβ1 | none | none |
| **LIVER-GENE (≥ 2FOLD)** | none | *Fn1, Alox5, Nos2*,*Olr1, Ptgs1* | *Fn1*, *Il1a,* | *Olr1, Ccl2*, *Cd40, Alox5, Lta4h, Nos2, Tgfb1* | none | *Edn1, Il6, Alox5, Ccl2, Cxcl5*. |
| **LIVER-PROTEIN (≥ 2FOLD)** | VEGF, IL1α, IL2, RETN | None | TGFβ, | SELL | SELL, TNFR1 | none |
| **AORTA-GENE (≥ 2FOLD)** | *S100a8,* | *Fga, Fgb, Fgg, Itgam, Lgals3, Gclc, Cxcl5* | *Edn1, Fga, Fgb, Fgg, Cxcl5, Selp, Lcn2, Saa3, Il10, Alox5, Emr1, Ncf1, Ncf2, Cxcl2, Icam1* | none | *Cxcl5,Csf2 ,Spp1* | none |

**Table S2:** BAL cell differentials are shown in counts 4 and 24 hrs after clean air (Contol) or carbon nanoparticle (CNP) inhalation. Values measured out of range were defined as: <OOR. Macro: macrophages, Lympho: lymphocytes, Neutro: neutrophil granulocyte, EOS: eosinophil. Means ± SEM, Inhalation : n=8; infusion: n=6; * p≤0.05.

|  |  |  | **Inhalation** | |  |  |
| --- | --- | --- | --- | --- | --- | --- |
|  |  | **4 h** |  |  | **24h** |  |
| **BAL cells** | **control** | **CNP** | **p** | **control** | **CNP** | **p** |
| Total cells (x10E06) | 0.58 ± 0.07 | 0.68 ± 0.09 | NS | 0.58 ± 0.07 | 0.49 ± 0.03 | NS |
| **Macrophages**  **(x10E06)** | **0.55 ± 0.01** | **0.67 ± 0.00** | ***↑** | 0.56 ± 0.00 | 0.47 ± 0.00 | NS |
| **Lymphocytes**  **(x10E03)** | **10.78 ± 0.76** | **9.20 ± 0.67** | ***↓** | **15.40 ± 0.89** | **8.15 ± 0.92** | ***↓** |
| **Neutrophils**  **(x10E03)** | **5.13 ± 0.60** | **1.50 ± 0.47** | ***↓** | **2.54 ± 0.80** | **10.15 ± 2.53** | ***↑** |
| Eosinophils (x10E03) | 0.41 ± 0.230 | 0.43 ± 0.262 | NS | <OOR | <OOR | NS |

**Table S3: BAL cytokine concentrations:** Protein markers in BAL samples of mice 4h and 24 h after clean air (Control) or carbon nanoparticle (CNP) inhalation. Values measured out of range were defined as: <OOR. CCL2: Monocyte chemoattractant protein-1 (MCP-1), CXCL1: Growth-regulated alpha protein (KC), G-CSF: Granulocyte colony stimulating factor, GM-CSF: Granulocyte-macrophage colony stimulating factor 2, IFN-gamma: Interferon gamma, IL: Interleukin, TNF-alpha: Tumor necrosis factor alpha. Mean ± SEM, * p≤0.05. n=8

| **Analytes** |  |  |  | **Inhalation** |  |  |  |
| --- | --- | --- | --- | --- | --- | --- | --- |
|  |  | **4h** | | | **24h** | | |
|  | **Conc.** | **Control** | **CNP** | **P** | **Control** | **CNP** | **P** |
| **CCL2** | pg/ml | 9.3 ± 3.7 | 12.6 ± 7.0 |  | **18.3 ± 4.1** | **1.8 ± 1.8** | ***↓** |
| **CXCL1** | pg/ml | **5.8 ± 1.3** | **2.2 ± 0.3** | ***↓** | **2.4 ± 0.2** | **10.9 ± 1.5** | ***↑** |
| G-CSF | pg/ml | 0.2 ± 0.05 | 0.1 ± 0.05 |  | 0.1 ± 0.04 | 0.2 ± 0.05 |  |
| GM-CSF | pg/ml | <OOR | <OOR |  | <OOR | <OOR |  |
| IFN-gamma | pg/ml | 1.3 ± 0.4 | 2.0 ± 1.0 |  | 2.2 ± 0.6 | 1.1 ± 0.5 |  |
| **IL-1 alpha** | pg/ml | **2.2 ± 0.2** | **1.2 ± 0.1** | ***↓** | **2.2 ± 0.2** | **1.5 ± 0.1** | ***↓** |
| IL-1 beta | pg/ml | <OOR | <OOR |  | <OOR | <OOR |  |
| IL-2 | pg/ml | 1.3 ± 0.2 | 1.0 ± 0.2 |  | 1.3 ± 0.2 | 1.3 ± 0.2 |  |
| IL-6 | pg/ml | 0.8 ± 0.2 | 0.6 ± 0.3 |  | 0.3 ± 0.2 | 0.3 ± 0.2 |  |
| IL-10 | pg/ml | <OOR | <OOR |  | <OOR | <OOR |  |
| **IL-12(p40)** | pg/ml | **23.9 ± 1.3** | **17.0 ± 1.1** | ***↓** | 18.7 ± 1.5 | 14.2 ± 1.2 |  |
| IL-13 | pg/ml | 5.4 ± 1.8 | 4.2 ± 2.0 |  | 7.2 ± 1.9 | 7.6 ± 2.4 |  |
| IL-17 | pg/ml | <OOR | <OOR |  | <OOR | <OOR |  |
| TNF-alpha | pg/ml | <OOR | <OOR |  | <OOR | <OOR |  |
| **Total protein** | μg/ml | **95.9 ± 8.4** | **136.3 ± 6.2** | ***↑** | 106.5 ± 4.2 | 106.5 ± 9.9 |  |

**Table S4: Gene annotation and primer sequences of the transcripts analyzed:** List of the 59 transcripts analyzed by quantitative RT-PCR with respective primer pairs. Expression of *Hprt1* expression served as control.

| **RefSeq** | **Gene Name** | **Entrez ID** | **Symbol** | **Primer Sequence - forward** | **Primer Sequence - reverse** |
| --- | --- | --- | --- | --- | --- |
| NM_207624 | Angiotensin I converting enzyme (peptidyl-dipeptidase A) 1 | 11421 | ***Ace*** | ctcagcctgggacttcta | cttgggggtagacactga g |
| NM_009662 | arachidonate 5-lipoxygenase | 11689 | ***Alox5*** | agagcagggagaagctgt | cacggtctggtagag tat cc |
| NM_009696 | apolipoprotein E | 11816 | ***Apoe*** | ggtcacattgctgacagg | agttctgcagctcttcct |
| NM_011333 | chemokine (C-C motif) ligand 2 | 20296 | ***Ccl2*** | cttctgggcctgctgttc a | ccagcctactcattgggatca |
| NM_027852 | CD40 antigen | 21939 | ***Cd40*** | gtcatctgtggtttaaagtcc ca | agagaaaca ccc cgaaaatgg |
| NM_011616 | CD40 ligand | 21947 | ***Cd40lg*** | ctcaaactctgaacagtgcgc t | ggcaggtcctaactg act tgc t |
| NM_009853 | CD68 antigen | 12514 | ***Cd68*** | cctcca ccc tcgcctagtc | ttgggtataggattcggatttga |
| NM_009969 | colony stimulating factor 2 (granulocyte- macrophage) | 12981 | ***Csf2*** | gccatcaaagaagccctg | gcgggtctgcacacatgt ta |
| NM_008176 | chemokine (C-X-C motif) ligand 1 | 14825 | ***Cxcl1*** | ccgaagtca tag ccacac | gtgccatca gag cag tct |
| NM_009140 | chemokine (C-X-C motif) ligand 2 | 20310 | ***Cxcl2*** | ctgttgtggccagtgaac | gcccttgagagtggc tat |
| NM_009141 | chemokine (C-X-C motif) ligand 5 | 20311 | ***Cxcl5*** | ccc tacggtggaagt cat | cttcactggggt cag agt |
| NM_010104 | endothelin 1 | 13614 | ***Edn1*** | ttc cag gtccaagcgttcct | gcttgg cag aaattc cag ca |
| NM_010130 | EGF-like module containing, mucin-like, hormone receptor-like sequence 1 | 13733 | ***Emr1*** | ctctgtggt ccc accttc at | gat ggccaaggatctgaa aa |
| NM_010171 | coagulation factor III | 14066 | ***F3*** | gag caatggaagagtttcct | gca cag agatatgga cag |
| NM_010196 | fibrinogen, alpha polypeptide | 14161 | ***Fga*** | agggtcacctgcctcatctt | aca act cttgggccacgt ac |
| NM_181849 | fibrinogen, B beta polypeptide | 110135 | ***Fgb*** | tgaggcatc tat ggctgc t | tgtcaa cag gtc gat gac ca |
| NM_133862 | fibrinogen, gamma polypeptide | 99571 | ***Fgg*** | agc ccc cgagtttccttctt | ggtggttgggcagaa act ac |
| NM_010233 | fibronectin 1 | 14268 | ***Fn1*** | acgtgcctt tag cacagagg | ccc agagccatcttgttttc |
| NM_010295 | glutamate-cysteine ligase, catalytic subunit | 14629 | ***Gclc*** | gag acccactca ccc ttt | tca ccc tag tgagcagta cc |
| NM_008181 | glutathione S-transferase, alpha 1 (Ya) | 14857 | ***Gsta1*** | aggaga gag ccc tgattg | ctgttg ccc acaagg tag |
| NM_010442 | hemeoxygenase (decycling) 1 | 15368 | ***Hmox1*** | aggtgatgctga cag agg | gtgtctgggatgagc tag tg |
| NM_013556 | hypoxanthine guanine phosphoribosyltransferase 1 | 15452 | ***Hprt1*** | gttggatacaggccagactttgt | cacagg act agaacacctgc |
| NM_010479 | heat shock protein 1A | 193740 | ***Hspa1a*** | gacaagtcg gag aacgtg | gag tag gtggtgaaggtctg |
| NM_010493 | intercellular adhesion molecule | 15894 | ***Icam1*** | ccg cag gtccaattcacact | tccagccgaggaccatac ag |
| NM_008337 | interferon gamma | 15978 | ***IFNg*** | tcaagtggcata gat gtggaagaa | tggctctgcaggattttcatg |
| NM_010548 | interleukin 10 | 16153 | ***Il10*** | tctgtc tag gtcctggagtc | ggagcaggtgaagagtga |
| NM_010554 | interleukin 1 alpha | 16175 | ***Il1a*** | agcgctcaaggagaagac | ctgtca tag agggcagtc c |
| NM_008361 | interleukin 1 beta | 16176 | ***Il1b*** | caaccaacaagt gat attctc cat g | gat ccacactctccagctgca |
| NM_031168 | interleukin 6 | 16193 | ***Il6*** | gccagagtcctt cag aga g | ccactccttctgtgactc c |
| NM_001082960 | integrin alpha M | 16409 | ***Itgam*** | cctcaagggcaacctatc | gacctcacatacgactcctg |
| NM_008491 | lipocalin 2 | 16819 | ***Lcn2*** | gaagaaccaaggagctgt | tcaatg cat tggtcggtg |
| NM_010700 | low density lipoprotein receptor | 16835 | ***Ldlr*** | cactgtggtagcagt gag tg | gct cat gtccttgcagtc |
| NM_010705 | lectin, galactose binding, soluble 3 | 16854 | ***Lgals3*** | gag ctacacatccctagc c | ctcaggaggatctgagactg |
| NM_008517 | leukotriene A4 hydrolase | 16993 | ***Lta4h*** | cag atggttacggctctg | agctcttgg tag gtgtggaca g |
| U360142 | lymphocyte antigen 6 complex, locus G | 16414 | ***Ly6g*** | tgg act ctcacagaagcaaag | gca gag gtcttccttccaaca |
| NM_013599 | matrix metallopeptidase 9 | 17395 | ***Mmp9*** | gaagtggggtttctgtcc | agccctcgaggtagc tat ac |
| NM_010876 | neutrophil cytosolic factor 1 | 17969 | ***Ncf1*** | ctgctgttgaagaggacgagatg | agccggtga tat ccc ctttcc |
| NM_010877 | neutrophil cytosolic factor 2 | 17970 | ***Ncf2*** | ctcgccagaacacactaa act ga | tccttcatgctttcttcggac ag |
| NM_010908 | nuclear factor of kappa light polypeptide gene enhancer in B cells inhibitor, beta | 18036 | *Nfkbib* | ctg cat ctagcagccatc | ggctct gag tgaggtagg ta |
| NM_010927 | nitric oxide synthase 2, inducible, macrophage | 18126 | ***Nos2*** | cctgtgagacctttgatg | cctatattgctgtggctc |
| NM_008713 | nitric oxide synthase 3, endothelial cell | 18127 | ***Nos3*** | gtagccaatgcagtgaag | cct cag agc cat acagaa |
| NM_138648 | oxidized low density lipoprotein (lectin- like) receptor 1 | 108078 | ***Olr1*** | agaggagtccca gag aga ac | cttgtggacaaggacctg |
| AK084527 | platelet/endothelial cell adhesion molecule 1 | 18613 | ***Pecam1*** | gtcttgtcg cag tat cag aatttc ag | tac cag gccgcttctcttga |
| NM_013737 | phospholipase A2, group VII | 27226 | ***Pla2g7*** | cctgcaagctggaattct cc | ccc attagatgccaagcc aa |
| NM_001081211 | platelet-activating factor receptor | 19204 | *Ptafr* | caacgagggcgactg gat t | gacacccaaaaaggccac act |
| NM_008969 | prostaglandin-endoperoxide synthase 1 | 19224 | ***Ptgs1*** | act ggtggatgccttctc | gta cag ctcctccaactc ag |
| NM_011198 | prostaglandin-endoperoxide synthase 2 | 19225 | ***Ptgs2*** | caacacctgagcggttac | gttccaggaggatggagt |
| NM_013650 | S100 calcium binding protein A8 (calgranulin A) | 20201 | ***S100a8*** | ccgtcttcaaga cat cgtttg a | gtagagggcatggtgatttcc t |
| NM_011315 | serum amyloid A 3 | 20210 | ***Saa3*** | gaagctggtcaagggtct | gtcagctcttgagtcctctg |
| NM_011345 | selectin, endothelial cell | 20339 | ***Sele*** | ggctgtaaaaggggctac | ctgtacccttccaca cag tc |
| NM_011347 | selectin, platelet | 20344 | ***Selp*** | tgcccaaaaggttcctgg a | gcagcc act tccttctgatt |
| NM_011346 | selectin, lymphocyte | 20343 | ***Sell*** | gcctga act tcacca gag | ctaggtcgaagctgg act c |
| NM_009263 | secreted phosphoprotein 1 | 20750 | ***Spp1*** | agctcagaggagaagctt | ctt cag aggaca cag cat |
| NM_011577 | transforming growth factor, beta 1 | 21803 | ***Tgfb1*** | tgacgtcactggagttgtacg | ggttcatgt cat ggatggtgc |
| NM_011580 | thrombospondin 1 | 21825 | ***Thbs1*** | ctggacttgctg tag gtt | aacaaagccagcgtagtc |
| NM_013693 | tumor necrosis factor | 21926 | ***Tnf*** | caccacgctcttctgtct | ggctacaggcttgtc act c |
| NM_011609 | tumor necrosis factor receptor superfamily, member 1a | 21937 | ***Tnfrsf1a*** | gat acagtctgcagg gag tg | cct gag tgagacacactt cc |
| NM_011693 | vascular cell adhesion molecule 1 | 22329 | ***Vcam1*** | gcctcgctaggttacaca | ggactg ccc tcctctagt at |
| NM_011708 | Von Willebrand factor homolog | 22371 | ***Vwf*** | agcctgaatccacgcttg a | ataacggcagttctg cag |

**Table S5: Haematological analysis** Haematological analysis of blood samples 4 and 24 hrs after clean air (Control) or carbon nanoparticle (CNP) inhalation and 4 hrs after sham (Control) or CNP infusion. RBC: red blood cells, WBC: white blood cells, Lympho: lymphocytes, Neutro: neutrophil granulocyte, EOS: eosinophil granulocyte, Baso: basophil granulocyte, Mono: monocytes, PLT: platelets, Large PLT: large platelets. Means ± SEM, * p<0.05. Inhalation: n=8/group; Infusion: n=6/group

|  | **Inhalation** | | | | | | **Infusion** | | |
| --- | --- | --- | --- | --- | --- | --- | --- | --- | --- |
|  | **4h** | | | **24h** | | | **4h** | | |
| **Peripheral blood cells** | **Control** | **CNP** | **P** | **Control** | **CNP** | **P** | **Control** | **CN** | **P** |
| RBC(x10E06/μL) | 9.27 ± 0.13 | 9.22 ± 0.05 |  | 9.10 ± 0.21 | 9.19 ± 0.08 |  | 10.42 ± 0.13 | 10.22 ± 0.41 |  |
| WBC(x10E03/μL) | 4.42 ± 0.55 | 4.03 ± 0.48 |  | 5.28 ± 0.77 | 5.09 ± 0.51 |  | 2.10 ± 0.14 | 3.05 ± 0.40 |  |
| **Lympho(x10E03/μL)** | 3.67 ± 0.47 | 3.39 ± 0.43 |  | 4.62 ± 0.75 | 4.16 ± 0.46 |  | **1.52 ± 0.07** | **1.92 ± 0.13** | ***↑** |
| **Neutro(x10E03/μL** | 0.46 ±0.09 | 0.48 ± 0.06 |  | **0.46 ± 0.09** | **0.75 ± 0.05** | ***↑** | 0.48 ± 0.13 | 0.99 ± 0.31 |  |
| EOS(x10E03/μL) | 0.229 ± 0.062 | 0.105 ± 0.032 |  | 0.139 ± 0.021 | 0.095 ± 0.006 |  | 0.075 ± 0.009 | 0.073 ± 0.007 |  |
| Baso(x10E03/μL) | 0.009 ± 0.003 | 0.004 ± 0.003 |  | 0.008 ± 0.003 | 0.008 ± 0.002 |  | 0.008 ± 0.003 | 0.012 ± 0.002 |  |
| **Mono(x10E03/μL)** | 0.040 ± 0.012 | 0.033 ± 0.005 |  | **0.040 ± 0.009** | **0.065 ± 0.010** | ***↑** | 0.007 ± 0.002 | 0.053 ± 0.027 |  |
| **PLT(x10E03/μL)** | **809.50 ± 86.95** | **1203.13 ± 54.84** | ***↑** | 868.50 ± 96.65 | 1069.38 ± 37.95 |  | 902.50 ± 62.56 | 909.67 ± 69.40 |  |
| **Large PLT(x10E03/μL)** | **1.88 ± 0.40** | **17.63 ± 2.71** | ***↑** | 4.25 ± 1.32 | 8.00 ± 1.59 |  | 22.33 ± 4.72 | 25.50 ± 5.60 |  |

**Table S6: Multiplex protein suspension array** Protein markers in plasma samples of mice 4 and 24 hrs after clean air (Control) or carbon nanoparticle (CNP) inhalation and 4 h after sham (Control) or CNP infusion. Values measured out of range were defined as: <OOR. CCL2: Monocyte chemoattractant protein-1 (MCP-1), CCL5: RANTES, CXCL1: Growth-regulated alpha protein (KC), E-selectin: Endothelial leukocyte adhesion molecule 1, G-CSF: Granulocyte colony stimulating factor, GM-CSF: Granulocyte-macrophage colony stimulating factor 2, IFN-gamma: Interferon gamma, IL: Interleukin, MMP9: Matrix metalloproteinase-9, sICAM-1: soluble Intercellular adhesion molecule 1, sVCAM-1: soluble Vascular adhesion molecule 1, TNF-alpha: Tumor necrosis factor alpha, total PAI-1: total Plasminogen activator inhibitor 1. Inhalation: n=8; infusion: n=6; Mean ± SEM, * p<0,05.

|  |  | **Inhalation** | | | | | | **Infusion** | | |
| --- | --- | --- | --- | --- | --- | --- | --- | --- | --- | --- |
|  |  | **4h** | | | **24h** | | | **4h** | | |
| **Analytes** | **Conc.** | **Control** | **CNP** | **P** | **Control** | **CNP** | **P** | **Control** | **CNP** | **P** |
| Adiponectin | μg/ml | 10.9 ± 2.8 | 13.1 ± 0.9 |  | 16.7 ± 2.2 | 10.3 ± 1.9 |  | 19.1 ± 1.4 | 21.4 ± 2.2 |  |
| **CCL2** | pg/ml | **39.8 ± 4.5** | **20.9 ± 2.1** | ***↓** | **35.9 ± 4.7** | **19.8 ± 2.6** | ***↓** | **4589.4 ± 297.9** | **2952.7 ± 770.1** | ***↓** |
| CCL5 | pg/ml | <OOR | <OOR |  | <OOR | <OOR |  | 2000.7 ± 144.7 | 1228.0 ± 307.8 |  |
| **CXCL1** | pg/ml | 22.2 ± 4.8 | 21.5 ± 2.4 |  | 21.5 ± 6.1 | 15.1 ± 2.0 |  | **2251.1 ± 169.7** | **1379.0 ± 391.8** | ***↓** |
| E-selectin | ng/ml | 0.0 ± 0.0 | 0.0 ± 0.0 |  | 5.9 ± 3.9 | 0.0 ± 0.0 |  | 207.8 ± 39.6 | 162.4 ± 27.0 |  |
| Fibrinogen | μg/ml | 66.1 ± 30.9 | 62.8 ± 24.2 |  | 53.3 ± 31.5 | 11.1 ± 3.4 |  | 853.6 ± 163.2 | 780.9 ± 96.1 |  |
| **G-CSF** | pg/ml | 28.8 ± 5.5 | 18.8 ± 1.4 |  | **24.7 ± 3.0** | **14.7 ± 0.9** | ***↓** | 2957.0 ± 24.3 | 2646.8 ± 187.7 |  |
| **GM-CSF** | pg/ml | 14.1 ± 2.1 | 8.5 ± 2.9 |  | **14.0 ± 2.4** | **6.0 ± 1.7** | ***↓** | **34.3 ± 2.6** | **26.3 ± 3.4** | ***↓** |
| IFN-gamma | pg/ml | 13.2 ± 5.3 | 2.2 ± 0.8 |  | 10.2 ± 5.4 | 1.5 ± 0.6 |  | 58.3 ± 4.5 | 66.3 ± 14.3 |  |
| **IL-1 alpha** | pg/ml | **42.2 ± 11.8** | **9.2 ± 2.5** | ***↓** | **18.7 ± 4.6** | **6.2 ± 0.4** | ***↓** | **6.4 ± 1.0** | **4.0 ± 1.1** | ***↓** |
| **IL-1 beta** | pg/ml | 151.0 ± 7.5 | 134.8 ± 5.1 |  | **144.2 ± 6.8** | **119.7 ± 5.0** | ***↓** | 133.7 ± 16.0 | 99.5 ± 13.5 |  |
| IL-2 | pg/ml | 1.1 ± 0.1 | 1.1 ± 0.2 |  | 1.5 ± 0.2 | 1.3 ± 0.3 |  | 20.4 ± 1.8 | 16.8 ± 2.6 |  |
| IL-4 | pg/ml | <OOR | <OOR |  | <OOR | <OOR |  | 15.7 ± 1.5 | 12.5 ± 1.2 |  |
| IL-5 | pg/ml | <OOR | <OOR |  | <OOR | <OOR |  | 82.6 ± 11.4 | 64.2 ± 9.2 |  |
| IL-6 | pg/ml | 5.1 ± 2.2 | 3.5 ± 1.4 |  | 5.7 ± 2.0 | 2.2 ± 1.0 |  | 2241.4 ± 477.1 | 1522.2 ± 463.4 |  |
| IL-10 | pg/ml | 0.6 ± 0.4 | 4.4 ± 4.5 |  | 7.3 ± 6.4 | 1.1 ± 1.0 |  | 337.2 ± 42.3 | 268.7 ± 53.5 |  |
| IL-12(p40) | pg/ml | 581.2 ± 143.5 | 513.8 ± 46.2 |  | 361.8 ± 88.9 | 322.0 ± 49.5 |  | 397.5 ± 266.1 | 749.8 ± 299.8 |  |
| IL-12(p70) | pg/ml | <OOR | <OOR |  | <OOR | <OOR |  | 14.7 ± 5.4 | 16.6 ± 6.4 |  |
| **IL-13** | pg/ml | 21.9 ± 2.3 | 15.5 ± 3.2 |  | 18.1 ± 3.3 | 21.6 ± 9.2 |  | **262.8 ± 18.3** | **193.3 ± 20.3** | ***↓** |
| IL-17 | pg/ml | 21.7 ± 14.7 | 8.4 ± 1.6 |  | 29.3 ± 12.7 | 9.8 ± 1.6 |  | 33.0 ± 4.7 | 53.4 ± 17.2 |  |
| MMP9 | ng/ml | 180.6 ± 33.1 | 136.2 ± 40.8 |  | 126.7 ± 11.2 | 162.1 ± 20.3 |  | 24.6 ± 7.2 | 49.3 ± 12.8 |  |
| sICAM1 | ng/ml | 16.9 ± 2.6 | 19.3 ± 3.1 |  | 19.4 ± 1.9 | 14.6 ± 2.9 |  | 99.6 ± 7.1 | 78.4 ± 13.2 |  |
| **sVCAM1** | ng/ml | 833.0 ± 134.6 | 880.7 ± 161.3 |  | **859.0 ± 62.8** | **581.6 ±66.1** | ***↓** | 1597.5 ± 211.9 | 1425.0 ± 154.0 |  |
| **TNF-alpha** | pg/ml | **101.2 ± 7.9** | **66.7 ± 11.1** | ***↓** | **87.7 ± 7.7** | **50.3 ± 6.0** | ***↓** | 670.3 ± 78.9 | 576.0 ± 121.7 |  |
| Total PAI-1 | ng/ml | 9.4 ± 2.7 | 6.5 ± 2.3 |  | 8.7 ± 3.3 | 9.0 ± 4.1 |  | 173.2 ± 30.6 | 139.3 ± 34.6 |  |

**Table S7:Multiplex protein expression analysis** Protein expression analysis (pg/ml) of lung, heart and liver tissue 4 and 24 h after clean air (Control) or carbon nanoparticle (CNP) inhalation and 4 h after sham (Control) or CNP infusion. CCL2: Monocyte chemoattractant protein-1 (MCP-1), CCL3: MIP-1-alpha, CCL4: MIP-1-beta, CRP: C-reactive protein. CXCL1: Growth-regulated alpha protein (KC), CXCL2: Macrophage inflammatory protein 2 (MIP2), CXCL12: Stromal cell-derived factor 1 (SDF-1), E-selectin: Endothelial leukocyte adhesion molecule 1, GM-CSF: Granulocyte-macrophage colony stimulating factor 2, ICAM-1: Intercellular adhesion molecule 1, IFN-gamma: Interferon gamma, IL: Interleukin, L-selectin: Leukocyte adhesion molecule 1 (CD62L antigen), MMP2: Matrix metalloproteinase-2, MMP9: Matrix metalloproteinase-9, PDGF-AA: Platelet-derived growth factor A chain (dimer), PDGF-BB: Platelet-derived growth factor B chains (dimer), P-selectin: Leukocyte-endothelial cell adhesion molecule 3 (CD62P antigen), TGF-beta-1: Transforming growth factor beta-1, TNF-alpha: Tumor necrosis factor alpha, TNF-RI: Tumor necrosis factor receptor superfamily member 1A, VCAM-1: Vascular adhesion molecule 1, VEGF: Vascular endothelial growth factor. Samples were pooled from 4 animals/experimental group for gene and protein analysis.

|  |  |  | **Inhalation** | | | | **Infusion** | |
| --- | --- | --- | --- | --- | --- | --- | --- | --- |
|  |  |  |  | **4h** |  | **24h** |  | **4h** |
|  | **Analytes** | **Conc** | **Control** | **CNP** | **Control** | **CNP** | **Control** | **CNP** |
|  | Adiponectin | pg/ml | 54265 | 67395 | 74830 | 87545 | 63080 | 62510 |
|  | CCL2 | pg/ml | 83.4 | 91.4 | 119.8 | 114.8 | 1480 | 1215 |
|  | CCL3 | pg/ml | 9.6 | 18 | 21.2 | 23 | 126.6 | 126.2 |
|  | CCL4 | pg/ml | 17 | 30 | 32 | 49 | 205 | 135.2 |
|  | CRP | pg/ml | 51.2 | 38.8 | 50.2 | 44.8 | 258.2 | 211.6 |
|  | CXCL1 | pg/ml | 101.4 | 142.2 | 128.2 | 177 | 2895 | 2190 |
|  | CXCL2 | pg/ml | 152 | 186 | 186.8 | 183.4 | 903 | 812.4 |
| **Lung tissue** | CXCL12 | pg/ml | 290.4 | 992.2 | 852.6 | 1007.4 | 456.8 | 278.2 |
|  | E-selectin | pg/ml | 29.4 | 35.2 | 27.8 | 35.4 | 576.2 | 433 |
|  | GM-CSF | pg/ml | 6.6 | 20.4 | 11.4 | 26.8 | 12 | 10.2 |
|  | ICAM1 | pg/ml | 13628 | 50815 | 43720 | 59595 | 51390 | 46775 |
|  | IFN-gamma | pg/ml | 657.2 | 983.4 | 830.4 | 928 | 946.8 | 825.2 |
|  | IL-1 alpha | pg/ml | 83.2 | 141.6 | 104 | 138.4 | 135.4 | 111.2 |
|  | IL-1 beta | pg/ml | 28.6 | 61.8 | 47.6 | 80.2 | 115 | 109.4 |
|  | IL-1 ra | pg/ml | 293.8 | 456.6 | 439.8 | 561.8 | 423.4 | 329 |
|  | IL-2 | pg/ml | 27.8 | 42.8 | 30.6 | 53 | 36.6 | 25.4 |
|  | IL-6 | pg/ml | 80.2 | 167.6 | 73 | 157.8 | 1676.2 | 860.2 |
|  | IL-10 | pg/ml | 20.2 | 70.8 | 26.2 | 65.4 | 39.6 | 38 |
|  | IL-12(p40) | pg/ml | 1.2 | 2.2 | 1.6 | 1.8 | 5 | 5 |
|  | IL-13 | pg/ml | 70.6 | 122 | 112 | 169 | 160.4 | 130.2 |
|  | IL-17 | pg/ml | 25.6 | 51.4 | 41.8 | 74 | 65 | 58.8 |
|  | L-selectin | pg/ml | 9670 | 14340 | 16185 | 17885 | 10405 | 11075 |
|  | MMP2 | pg/ml | 84110 | 255655 | 122710 | 248325 | 135915 | 132105 |
|  | MMP9 | pg/ml | 67245 | 97005 | 117100 | 151590 | 201290 | 214735 |
|  | Osteopontin | pg/ml | 1230.4 | 1956.6 | 2343.4 | 2411.2 | 7202 | 5490 |
|  | PDGF-AA | pg/ml | 4.8 | 5.2 | 5.8 | 4.8 | 5.8 | 5.6 |
|  | PDGF-BB | pg/ml | 151.8 | 320.4 | 268.6 | 369.6 | 208.6 | 198.4 |
|  | P-selectin | pg/ml | 21915 | 40300 | 28600 | 39400 | 47100 | 36717 |
|  | Resistin | pg/ml | 305.4 | 645 | 445 | 785 | 349.8 | 440 |
|  | TGF-beta-1 | pg/ml | 17390 | 1295 | 16215 | 13045 | 6046.4 | 6748 |
|  | TNF-alpha | pg/ml | 14.8 | 75.2 | 37 | 72.4 | 79 | 67.4 |
|  | TNFR-I | pg/ml | 268.8 | 540 | 445 | 610 | 151.2 | 166.6 |
|  | VCAM1 | pg/ml | 4975.2 | 8315 | 7842.6 | 9210 | 7320 | 5639.8 |
|  | VEGF | pg/ml | 58.4 | 121.8 | 84.6 | 130.8 | 110.2 | 108.4 |
|  |  |  | **Inhalation** | | | | **Infusion** | |
|  |  |  |  | **4h** |  | **24h** |  | **4h** |
|  | **Analytes** | **Conc** | **Control** | **CNP** | **Control** | **CNP** | **Control** | **CNP** |
|  | CCL3 | pg/ml | 7.2 | 8.8 | 8 | 11 | 25.6 | 21.2 |
|  | CCL4 | pg/ml | 5 | 5.4 | 4 | 6.6 | 24.2 | 21 |
|  | CRP | pg/ml | 111.8 | 75.4 | 91 | 79.8 | 261.4 | 256 |
|  | CXCL1 | pg/ml | 42.2 | 55 | 35.6 | 53.4 | 461.4 | 333 |
|  | CXCL2 | pg/ml | 85 | 124.2 | 76.4 | 93.6 | 193 | 174.2 |
|  | CXCL12 | pg/ml | 989.4 | 847.8 | 873.4 | 1058.4 | 977 | 867 |
|  | E-selectin | pg/ml | 55.2 | 61.6 | 49.6 | 48.4 | 1413.4 | 884.2 |
| **Heart tissue** | GM-CSF | pg/ml | 8.4 | 8.4 | 7.2 | 13.2 | 15.8 | 17.4 |
|  | ICAM1 | pg/ml | 1549.2 | 1323.4 | 939.2 | 1098.4 | 5873.8 | 4437.6 |
|  | IFN-gamma | pg/ml | 362.8 | 256.8 | 336.8 | 347 | 812.4 | 687.4 |
|  | IL-1 alpha | pg/ml | 32 | 19.2 | 28.4 | 28.2 | 92.4 | 75.4 |
|  | IL-1 beta | pg/ml | 25 | 18.8 | 28 | 25.6 | 50.8 | 56 |
|  | IL-1 ra | pg/ml | 74.2 | 97 | 56.4 | 92.4 | 170.8 | 146.4 |
|  | IL-2 | pg/ml | 20.6 | 13.4 | 17 | 21 | 41 | 39.6 |
|  | IL-6 | pg/ml | 117 | 136.4 | 103.2 | 187.2 | 665.6 | 456.8 |
|  | IL-10 | pg/ml | 101.8 | 97 | 87 | 162.2 | 115.6 | 101.4 |
|  | IL-12(p40) | pg/ml | 0.8 | 1 | 0.8 | 1.2 | 2.4 | 2.2 |
|  | IL-13 | pg/ml | 55.8 | 56 | 40.6 | 82.8 | 94.8 | 124.8 |
|  | IL-17 | pg/ml | 18.4 | 17.4 | 18.4 | 33.2 | 40.6 | 57.8 |
|  | L-selectin | pg/ml | 2723.6 | 2239 | 2452.6 | 2372 | 6110 | 5925 |
|  | MMP2 | pg/ml | 20747.4 | 25480 | 21848.2 | 20624.4 | 52820 | 63926 |
|  | MMP9 | pg/ml | 2129.6 | 2521.4 | 2841.6 | 2864.4 | 12376.8 | 10330 |
|  | Osteopontin | pg/ml | 470.8 | 924.6 | 297.8 | 436.8 | 1242 | 1532.6 |
|  | PDGF-AA | pg/ml | 2.2 | 2.6 | 2.6 | 1.8 | 3.4 | 3 |
|  | PDGF-BB | pg/ml | 95.8 | 104.2 | 99.2 | 137.8 | 160.6 | 185.8 |
|  | P-selectin | pg/ml | 8315 | 3515 | 11325 | 1515 | 11090 | 7835 |
|  | Resistin | pg/ml | 705 | 580 | 895 | 490 | 675 | 630 |
|  | TGF-beta-1 | pg/ml | 789.2 | 742.2 | 1587.8 | 525.1 | 1550.2 | 906.2 |
|  | TNF-alpha | pg/ml | 57.4 | 57.4 | 69.4 | 79.2 | 95.8 | 91.2 |
|  | TNFR-I | pg/ml | 38.6 | 39 | 39.8 | 38 | 33.2 | 37.8 |
|  | VCAM1 | pg/ml | 730.8 | 629.4 | 560 | 623.8 | 2186.8 | 1971.4 |
|  | VEGF | pg/ml | 17.4 | 15.6 | 16 | 21.8 | 34.6 | 35.2 |
|  |  |  | **Inhalation** | | | | **Infusion** | |
|  |  |  |  | **4h** |  | **24h** |  | **4h** |
|  | **Analytes** | **Conc** | **Control** | **CNP** | **Control** | **CNP** | **Control** | **CNP** |
|  | Adiponectin | pg/ml | 22591 | 22705 | 24910 | 22376 | 14797 | 20930 |
|  | CCL2 | pg/ml | 34.6 | 40 | 44.2 | 33.8 | 1045 | 1140 |
|  | CCL3 | pg/ml | 29.8 | 43.2 | 40.4 | 37.2 | 70 | 78.4 |
|  | CCL4 | pg/ml | 15.4 | 22 | 19.4 | 21.8 | 58 | 67 |
|  | CRP | pg/ml | 263 | 402.2 | 398.4 | 329.2 | 527.6 | 656.8 |
|  | CXCL1 | pg/ml | 132.8 | 166 | 157.4 | 173.4 | 877.2 | 904.6 |
|  | CXCL2 | pg/ml | 135.4 | 180.8 | 158.4 | 155.6 | 301 | 364 |
| **Liver tissue** | CXCL12 | pg/ml | 1487 | 2656.4 | 2784.2 | 2393.8 | 1384 | 1452.4 |
|  | E-selectin | pg/ml | 38.4 | 75.8 | 86.6 | 69.6 | 1068.8 | 1153.8 |
|  | GM-CSF | pg/ml | 72.8 | 90.4 | 97.4 | 90.8 | 51.2 | 70.4 |
|  | ICAM1 | pg/ml | 1065.8 | 1381.6 | 1649.2 | 1208.2 | 7084.2 | 8046.6 |
|  | IFN-gamma | pg/ml | 773.2 | 1421.4 | 1379.4 | 1008.4 | 1399.4 | 1663.2 |
|  | IL-1 alpha | pg/ml | 86.6 | 215 | 210.4 | 138.8 | 929.4 | 786.6 |
|  | IL-1 beta | pg/ml | 120.8 | 236.6 | 228 | 156 | 157.6 | 200.2 |
|  | IL-1 ra | pg/ml | 254.8 | 376.4 | 414 | 342.2 | 718.2 | 627.8 |
|  | IL-2 | pg/ml | 50.4 | 119.6 | 119.8 | 79.8 | 88.6 | 118.4 |
|  | IL-6 | pg/ml | 568.8 | 666.4 | 858.6 | 656.4 | 1576 | 1443.6 |
|  | IL-10 | pg/ml | 721.2 | 982.2 | 1373.4 | 1195.8 | 865.6 | 917.6 |
|  | IL-12(p40) | pg/ml | 2.4 | 4.2 | 4 | 3.2 | 19.8 | 16.4 |
|  | IL-13 | pg/ml | 202.2 | 232 | 221 | 233 | 164.2 | 212.8 |
|  | IL-17 | pg/ml | 124 | 152.6 | 148.6 | 149.6 | 83.2 | 118.8 |
|  | L-selectin | pg/ml | 1159.4 | 1233.2 | 1683.4 | 759.8 | 1943.4 | 2922.8 |
|  | MMP2 | pg/ml | 10159 | 15099 | 13202.8 | 10106.8 | 52777 | 74285 |
|  | MMP9 | pg/ml | 3195.4 | 4546.8 | 5945.6 | 4080.4 | 30775 | 40790 |
|  | Osteopontin | pg/ml | 1100 | 1945.2 | 1845.4 | 1789 | 2211.8 | 2697.2 |
|  | PDGF-AA | pg/ml | 2.4 | 3.6 | 3 | 3.4 | 4.2 | 5.2 |
|  | PDGF-BB | pg/ml | 427.6 | 713.2 | 788.4 | 589.4 | 208 | 290.4 |
|  | P-selectin | pg/ml | 1655 | 2465 | 2575 | 1890 | 5195 | 7100 |
|  | Resistin | pg/ml | 86.6 | 191.6 | 136 | 156.6 | 221.6 | 262.2 |
|  | TGF-beta-1 | pg/ml | 631 | 572.8 | 279.4 | 1102.2 | 781.4 | 742 |
|  | TNF-alpha | pg/ml | 230.4 | 404.8 | 568.6 | 321 | 337.2 | 441.4 |
|  | TNFR-I | pg/ml | 54.4 | 85.2 | 81.6 | 69 | 31.6 | 50.8 |
|  | VCAM1 | pg/ml | 708 | 1330.8 | 1215.8 | 1142 | 1696.6 | 2076.2 |
|  | VEGF | pg/ml | 55.8 | 116.2 | 114.4 | 89.2 | 44.8 | 63.8 |

**Table S8:** Animal distribution for each experiment. **CNP:** Carbon nanoparticle; **FACS:** Fluorescence automated cell sorter

| **INHALATION**  4h-control (n=16) and 4h-CNP (n=16)  24h-control (n=16) and 24h-CNP (n=16) | **INTRA ARTERIAL INFUSION**  4h-control (n=6) and 4h-CNP (n=6) |
| --- | --- |
| Distribution within each experimental group:   - Bronchoalveolar lavage (cell differentials, cytokine analysis): n=8 - Blood collection for hematology (retro-orbital) and plasma protein analysis: n=8 - Blood collection for FACS: n=8 - Lung, heart, liver, and aortic tissue collection for gene expression and protein analysis: n=4 - Lung Histology: n=4 | Distribution within each experimental group:   - Bronchoalveolar lavage: n=6; (cell differentials) - Blood collection for hematology (retroorbital) and plasma protein isolation: n=6 - Blood collection for FACS: n=6 - Lung, heart, liver, and aortic tissue collection for gene expression analysis: n=6 - Lung, heart, liver, and aortic tissue collection for protein expression analysis: n=6 |

**Table S9: Comparison of the properties form Printex 90 (IAI) and Palas spark-discharge (Inhalation) carbon nanoparticles (CNP).**

|  | **Ptx90**  **CNP** | **Palas**  **CNP** | **rel. to Palas** | **References** |
| --- | --- | --- | --- | --- |
| **BET (cm^2^/g)** | 300 | 800 | 38% | Stoeger et al. 2009 |
| **Primary particle size (nm)** | 14 ±2 | 10 ±2 | 140% | Stoeger et al. 2009 |
| **Organic carbon (%)** | **2** | **<5** | >40% | Stoeger et al. 2009, Matuschek et al. 2007; Frampton et al. 2004) |
|  |  |  |  |  |
| **Oxidative Potency (OxPot)** |  |  |  |  |
| **Ascorbate consumption (nmol/µg)** | **0.28** | **0.84** |  | Stoeger et al. 2009 |
| **Ascorbate consumption (nmol/mm^2^)** | 0.09 | 0.10 | 88% |  |
|  |  |  |  |  |
| **8-Isoprostane generation (pg)** | **54.8** | **97.6** |  | Beck-Speier et al. 2005 |
| **8-Isoprostane generation (pg/mm^2^)** | 57 | 38 | 150% |  |
|  |  |  |  |  |
| **Inflammatory Potency (IEf)** |  |  |  |  |
| **IEf (%PMN/ug)** | **1.2** | **4.8** |  | Stoeger et al. 2009 |
| **IEf (%PMN/mm^2^)** | 0.40 | 0.60 | 67% |  |
|  |  |  |  |  |

**References**:

Beck-Speier I, Dayal N, Karg E, Maier KL, Schumann G, Schulz H, Semmler M, Takenaka S, Stettmaier K, Bors W, Ghio A, Samet JM, Heyder J. Oxidative stress and lipid mediators induced in alveolar macrophages by ultrafine particles. Free Radic Biol Med. 2005 Apr 15;38(8):1080-92.

Frampton MW, Utell MJ, Zareba W, Oberdörster G, Cox C, Huang LS, Morrow PE, Lee FE, Chalupa D, Frasier LM, Speers DM, Stewart J. Effects of exposure to ultrafine carbon particles in healthy subjects and subjects with asthma. Res Rep Health Eff Inst. 2004 Dec;(126):1-47.

Matuschek G, Karg E, Schröppel A, Schulz H, Schmid O. Chemical investigation of eight different types of carbonaceous particles using thermoanalytical techniques. Environ Sci Technol. 2007 Dec 15;41(24):8406-11.

Stoeger T, Takenaka S, Frankenberger B, Ritter B, Karg E, Maier K, Schulz H, Schmid O. Deducing in vivo toxicity of combustion-derived nanoparticles from a cell-free oxidative potency assay and metabolic activation of organic compounds. Environ Health Perspect. 2009 Jan;117(1):54-60.

Stoeger T, Reinhard C, Takenaka S, Schröppel A, Karg E, Ritter B, Heyder J, Schulz H Instillation of six different ultrafine carbon particles indicate a surface area threshold dose for acute lung inflammation in mice. Environ. Health Perspect. 2006 Mar; 114(3):328-333.
